# Ceramide and Related Molecules in Viral Infections

**DOI:** 10.3390/ijms22115676

**Published:** 2021-05-26

**Authors:** Nadine Beckmann, Katrin Anne Becker

**Affiliations:** Department of Molecular Biology, University of Duisburg-Essen, 45141 Essen, Germany; Katrin.Becker@uk-essen.de

**Keywords:** ceramide, acid sphingomyelinase, sphingolipids, lipid-rafts, α-galactosylceramide, viral infection, antiviral therapies, immunomodulation, SARS-CoV-2, HIV-1, IAV

## Abstract

Ceramide is a lipid messenger at the heart of sphingolipid metabolism. In concert with its metabolizing enzymes, particularly sphingomyelinases, it has key roles in regulating the physical properties of biological membranes, including the formation of membrane microdomains. Thus, ceramide and its related molecules have been attributed significant roles in nearly all steps of the viral life cycle: they may serve directly as receptors or co-receptors for viral entry, form microdomains that cluster entry receptors and/or enable them to adopt the required conformation or regulate their cell surface expression. Sphingolipids can regulate all forms of viral uptake, often through sphingomyelinase activation, and mediate endosomal escape and intracellular trafficking. Ceramide can be key for the formation of viral replication sites. Sphingomyelinases often mediate the release of new virions from infected cells. Moreover, sphingolipids can contribute to viral-induced apoptosis and morbidity in viral diseases, as well as virus immune evasion. Alpha-galactosylceramide, in particular, also plays a significant role in immune modulation in response to viral infections. This review will discuss the roles of ceramide and its related molecules in the different steps of the viral life cycle. We will also discuss how novel strategies could exploit these for therapeutic benefit.

## 1. Introduction

**Figure 1:**
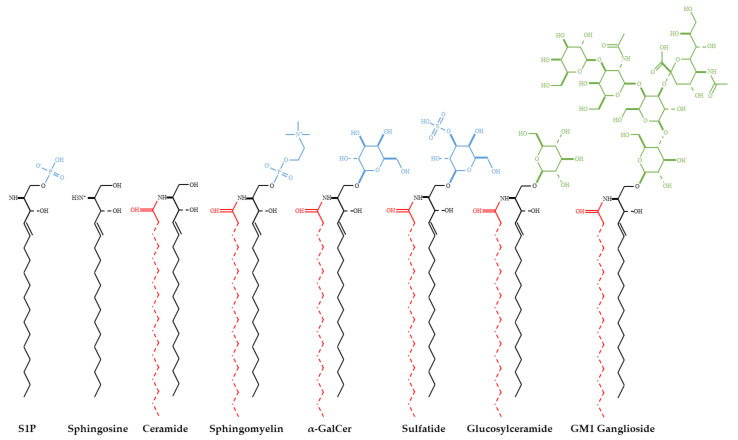
Sphingolipid structures. Ceramide consists of a sphingosine backbone (black) and one of several fatty acids (red), which differ in chain length and degree of saturation. Thus, the term “ceramide” actually describes a whole class of molecules. The addition of certain, invariant polar head groups (blue) results in sphingolipid classes that only vary in their underlying ceramide, whereas the addition of variable sugar groups (green) results in different glycosphingolipids. These are the most complex group, as the type and linkage of sugar residues added differ in addition to the fatty acid chain.

**Figure 2:**
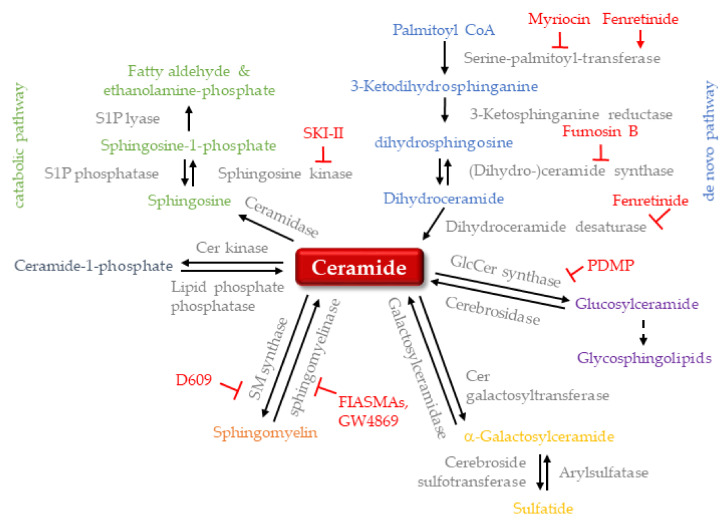
Sphingolipid metabolism. Ceramide is the central hub of sphingolipid metabolism. De novo synthesis (blue) starts with palmitoyl CoA, and the salvage pathway starts with conversion to sphingosine and ends with a fatty aldehyde and ethanolamine-phosphate (green). The synthesis of glycosphingolipids starts with the formation of glucosylceramide (purple). Other key pathways are phosphorylation to ceramide-1-phosphate (blue-gray), conversion to sphingomyelin (orange) and glycosylation to α-galactosylceramide and sulfatide (yellow). Inhibitors of ceramide-metabolizing enzymes are shown in red. Cer: ceramide; GlcCer: glucosylceramide; S1P: sphingosine 1-phosphate; SM: sphingomyelin.

Ceramide is an important lipid messenger that consists of a sphingosine backbone, which is acylated with one of several possible acyl coenzyme A molecules by a ceramide synthase [1]. Thus, the term “ceramide” technically comprises a whole class of molecules that differ in their acyl chain and can have different biological functions as a result. The attachment of phosphocholine to ceramide yields sphingomyelin (SM) species, which differ in their acyl chains just like the underlying ceramides. However, it is still unclear if this is also linked to unique functions [2]. The addition of sugar residues to ceramide yields glycosphingolipids (GSL), which introduces an even greater level of complexity, as GSLs vary not only in their acyl chain but also in the order and type of sugar residues attached [2]. Figure 1 summarizes sphingolipid structures, and Figure 2 shows the metabolic pathway. 

SM is the most abundant sphingolipid in eukaryotes and a major component of cell membranes. Sphingomyelinases catalyze the breakdown of SM to ceramide and phosphorylcholine. Acid sphingomyelinase (ASM) is one of three such lipid hydrolases in humans [3]. Generally considered a lysosomal enzyme, ASM can, however, translocate to the plasma membrane and generate ceramide at the extracellular cell surface. This results in the formation of lipid microdomains called ceramide-enriched plasma membrane platforms, which are often crucial for ASM/ceramide-mediated signaling pathways [4]. This mechanism was first described for CD95-death induced signaling complex formation [5], but has since been demonstrated to occur for a large number of stimuli, including a number of pathogens [6].

In line with its role in membrane microdomains, ceramide has also been attributed key roles in internal membranes. The tight packing of ceramide molecules in response to sphingomyelinase activation causes a negative curvature. Depending on where in the lipid bilayer ceramide generation occurs, this can promote inward or outward vesiculation and promote endocytosis, vesicle shedding, etc. [7]. 

Taken together, it is no surprise that ceramide has been attributed a role in essentially all steps of the viral life cycle, from viral entry into the host cell over replication to the release of new virions. In this review, we will summarize the current knowledge regarding the involvement of ceramide and ceramide-containing sphingolipids at each step. We will also point out potential therapeutic interventions and discuss the roles of sphingolipids in viral immune modulation and evasion. 

## 2. Viral Entry

The first stage in the viral life cycle is cellular entry, which entails the attachment of a virion to a host cell, penetration of the virus into the cell cytoplasm and uncoating, i.e., the shedding of the capsid [8]. Given the known effects of sphingolipids on the organization of membranes and their biophysical properties [7,9,10] it is not surprising that they play a role in viral entry. While we present the reported data according to their role in the respective viral entry step, it has to be noted that, based on the experiments performed, it is not always possible to draw a clear distinction between the role of sphingolipids in viral attachment vs. penetration into host cells. Additionally, these roles do not have to be mutually exclusive. 

### 2.1. Viral Attachment

Several viruses use GSLs with a ceramide core as host cell receptors or co-receptors for infection. For instance, rotavirus binds different glycolipids and gangliosides [11,12]. GSLs may simply serve as receptors for initial viral attachment directly. Alternatively, they may serve as platforms for viral attachment: sphingolipids are organized in functional microdomains within the membrane, and these are associated with specific membrane proteins that could serve as viral receptors or co-receptors. These membrane microdomains can behave as moving platforms, allowing the recruitment of co-receptors after the initial virus–receptor interaction [13]. Microdomains could thus either stabilize the attachment of the virus to the cell surface through multiple low affinity interactions between the viral glycoprotein and lipid headgroups and/or convey the virus to an appropriate co-receptor by clustering and activating receptor molecules [13]. A prominent example that led to this hypothesis is human immunodeficiency virus (HIV-1). Principally, the HIV-1 envelope glycoprotein binds to CD4, the primary receptor, and then to a co-receptor (CCR5 or CXCR4), triggering large structural rearrangements of the glycoprotein and initiating membrane fusion [14]. However, the infection of CD4-negative cell lines have been described, leading to the identification of galactosylceramide (Galβ1-1′Cer, GalCer) [15,16,17,18,19,20], globotriaosylceramide (Gb3) and ganglioside GM3 [13,21,22,23] as alternate entry (co-)receptors and to the suggestion that GSL microdomains stabilize HIV-1 attachment to the cell surface and facilitate co-receptor recruitment [13,24,25]. 

Other viruses potentially using similar sphingolipid-domain-mediated entry pathways are influenza A virus (IAV), which binds GalCer [26] and N-acetylneuraminyllactosylceramide (GM3-NeuAc) [27], norovirus GII.4, which recognizes GalCer [28], rubella virus, which requires SM and cholesterol [29], and rhinovirus, which requires ceramide-enriched platforms [30,31,32]. Table 1 provides a summary of viruses reported to utilize sphingolipids for cell entry. 

Direct interaction between a virus and a specific host cell sphingolipid has not been demonstrated for all of these cases. Some reports only demonstrate the colocalization of virions with lipid rafts and/or reduced viral attachment when lipid composition is altered experimentally. However, direct binding is not a necessary requirement for the role of sphingolipid microdomains in viral uptake—it is also conceivable that the virus receptor is a cell surface protein that preferentially localizes within domains and/or that only holds the conformation required for virus binding within specific lipid domains. An example is angiotensin-converting enzyme 2 (ACE2), the receptor for severe acute respiratory syndrome coronavirus (SARS-CoV) and SARS-CoV-2 [33], which is preferentially located in lipid rafts [34]. Another is CD300lf, a cell surface protein required for murine norovirus entry [35]; in the absence of serine palmitoyltransferase (and thus de novo sphingolipid synthesis), CD300lf is still expressed on the cell surface, but the conformation is altered in such a way that CD300lf is no longer recognized by murine norovirus [36]. In this manner, sphingolipid metabolism can regulate viral tropism even without affecting the expression of cell surface receptors. 

Rather than affecting the conformation of a host receptor protein, the interaction with sphingolipids can also be crucial for the conformation of viral proteins required for entry. For example, human parvovirus B19 (B19V) colocalizes with the GSL globotetraosylceramide (Gb4Cer) in lipid rafts [37]. The interaction triggers a conformational change in the capsid, exposing the N terminus of the capsid structural protein VP1, which is critical for virus internalization [38]. Rather than resulting in viral uptake directly, however, the majority of initially bound virions actually detach from Gb4Cer, and, due to the conformational change in VP1, then exhibit superior cell binding capacity and infectivity when added to uninfected cells [38].

### 2.2. Viral Penetration and Uncoating

After the initial attachment of the virus, the viral material has to be introduced into the host cell and the viral genome made accessible. *Uncoating* is often immediately linked to the uptake process [48]. Generally, virus uptake can occur through *fusion* at the plasma membrane, micropinocytosis or endocytosis. Viruses are not necessarily restricted to one pathway—for instance, SARS-CoV-2 can enter through plasma membrane fusion, as well as through endocytosis [33,49,50]. 

In measles virus (MV) infection, ceramide plays an important role in the intermediate steps between viral attachment and entry: the binding of MV to its receptor DC-SIGN (dendritic cell-specific intracellular adhesion molecule-3-grabbing non-integrin, CD209) causes the rapid activation of neutral sphingomyelinase (NSM) and ASM and the formation of ceramide-enriched membrane microdomains. This promotes the surface localization of CD150 from intracellular storage compartments along with ASM. CD150 clusters with DC-SIGN and promotes MV fusion with the plasma membrane [44,45]. 

Ebola virus (EBOV), which enters cells through micropinocytosis, has also been reported to result in ASM recruitment to the site of viral attachment, and ASM activity is required for EBOV infection [39]. A drug-combination screen identified two drug combinations that effectively blocked EBOV entry, and the drugs in the identified combinations were inhibitors of Niemann-Pick C1, acid sphingomyelinase and lysosomal calcium release [51]. EBOV was shown to bind to SM-rich regions in the plasma membrane and the depletion of SM strongly reduced infection [39]. As DC-SIGN also binds EBOV glycoproteins [52], it is possible that both MV and EBOV recruit ASM from the lysosome through a DC-SIGN-mediated signaling pathway. 

In contrast to the MV/CD150 example of ceramide generation leading to the surface localization of an entry factor, ceramide may also promote receptor internalization: in the context of hepatitis C virus (HCV) infection, sphingomyelinase treatment results in the internalization of CD81 [53]. CD81 is an essential post-attachment entry factor for HCV, and its internalization inhibits infection. 

For HIV-1, increasing cellular ceramide levels through the stimulation of de novo synthesis, the exogenous addition of ceramide or through the enzymatic cleavage of SM at the plasma membrane rendered cells resistant to infection by blocking membrane fusion [54]. An increase of ceramide levels in response to fenretinide treatment (N-(4-hydroxyphenyl)retamide, 4-HPR, synthetic retinoid derivative) did not alter HIV-1 receptor distribution but led to increased viral binding and endocytotic uptake [55]. Compared to fusion at the plasma membrane, endocytosis is a less productive method of infection for HIV-1 due to increased viral degradation [56]. In contrast, IAV exploits the endocytotic pathway for entry into the cells. Consequently, IAV infection was enhanced by fenretinide treatment [55]. 

Membrane rupture is a key entry mechanism for many non-enveloped viruses. Adenovirus lytic protein-VI pierces the membrane, stimulating a calcium-influx and lysosomal exocytosis. Subsequently, endocytosis occurs to maintain the cell surface area, which the adenovirus hijacks for cell entry: ASM is activated and re-located to the cell surface in response to the adenovirus, and ASM-knockdown reduces infection, whereas exogenous ceramide enhances protein-VI binding and membrane rupture [57]. Human norovirus [58], *Neisseria meningitis* (intracellular bacteria) [59,60] and *Trypanosoma cruzi* (intracellular parasite) [61] similarly commandeer calcium- and ASM-dependent cellular wound removal processes. 

In addition to a role in modulating endocytotic uptake, sphingolipids also play a role in viral endosomal escape, which is necessary for successful replication in the cytoplasm. In case of IAV, recognition of the host cell receptor by the viral envelope protein hemagglutinin (HA) triggers endocytosis. The low pH in the endolysosome triggers conformational changes in HA, leading to the insertion of the fusion peptide into the host membrane and formation of a fusion pore [62]. Sphingolipids have been reported to affect the growth of the fusion pore, with SM, lactosyl cerebroside and glucosyl cerebroside inhibiting full pore enlargement [63]. In apparent contrast to the enhanced IAV infectivity observed upon fenretinide-increased ceramide levels, treatment with exogenous sphingomyelinase impairs IAV infection [64]. However, fenretinide treatment does not simultaneously alter SM levels [65], indicating that, in the latter study, the inhibitory effect was due to the consumption of SM rather than the increase of ceramide. In line with this, the addition of exogenous SM enhances IAV infection [64]. 

Low pH also triggers the fusion of Semliki Forest virus (SFV) with endosomal membranes. In a model system, low-pH-induced membrane fusion is mediated by sphingolipids in the target membrane, with ceramide being the sphingolipid that is minimally required [66,67]. Similar to the mechanism described for HA, ceramide does not appear to play a structural role in SVF fusion, but rather acts as a co-factor by inducing the fusion-active conformation of the viral fusion protein [68]. 

Bile acids facilitate the endosomal escape of calciviruses by triggering ceramide formation by ASM. Inhibition of ASM results in the retention of porcine enteric calcivirus, feline calcivirus and murine norovirus in the endosomes and reduces viral replication [69]. The downstream effects of ceramide generation were not studied further, but as ceramide is a known activator of cathepsin proteases [70], the authors hint that ceramide generation may lead to the activation of cathepsin L, which cleaves the calcivirus capsid protein, enabling replication [71]. For a human norovirus strain (GII.3), however, blocking cathepsin activity had no effect on viral replication, whereas ASM inhibition did significantly reduce replication [58].

The fusion of the viral envelope with the endosomal membrane and nucleocapsid release does not have to be concomitant steps. Reports with vesicular stomatitis virus (VSV) and flaviviruses show that these can also occur successively. In these cases, the viral membrane fuses with intraendosomal vesicles first, releasing the viral nucleocapsid into their lumen. Subsequently, the back fusion of the intraluminal vesicles with the limiting outer membrane of late endosomes releases the nucleocapsid into the cytoplasm [72,73]. In contrast, this back-fusion appears to be a cellular mechanism to limit infection with herpes simplex virus 1 (HSV-1). In this case, acid ceramidase loaded intraluminal vesicles with sphingosine. The uptake of HSV-1 into multivesicular bodies resulted in binding to sphingosine-rich intraluminal vesicles, trapping HSV-1 and preventing infection [74].

### 2.3. Targeting Sphingolipids to Prevent Viral Entry

The interaction between a virus and its lipid (co-)receptor is a potential therapeutic target. The disruption of GSL synthesis through the inhibition of glucosylceramide synthase (GlcGerS) with 1-phenyl-2-decanoylamino-3-morpholino-propanol (PDMP) reduced rotavirus infectivity [75]. Anti-GalCer antibody treatment significantly reduces productive HIV-1 infections in vitro [76], and analogues of GalCer or Gb3 were shown to interfere with HIV-1 viral entry in the absence of significant toxicity [77,78,79,80,81]. Moreover, the effective analogs showed co-receptor independent inhibition, as they inhibited CXCR4-, CCR5- and dual tropic virus variants [77,81] and even the infection of CD4^-^ cells [78]. Similarly, vaccinia virus (a surrogate of variola virus) was also reported to bind to sulfatide, a natural GalCer analogue, inhibiting infection [82]. 

ASM activity results in the release of ceramide on the cell surface in response to SARS-CoV-2 [83,84,85]. Functional inhibitors of ASM (FIASMAs), including drugs newly identified as FIASMAs like ambroxol, inhibit SARS-CoV-2 infection, as did the neutralization or consumption of surface ceramide [83,84,85]. FIASMAs were also shown to inhibit infection with two circulating IAV strains [84], as well as with Japanese encephalitis virus (JEV) [86] and EBOV [39]. In light of the current SARS-CoV-2/COVID-19 pandemic, which, at the time of this review, has already taken 3.5 Mio lives worldwide [87], this has piqued interest in repurposing FIASMAs as potential drugs against SARS-CoV-2 [83,84,85,88,89,90,91]. A retrospective cohort study already reported a positive association between chronic FIASMA administration and reduced mortality in COVID-19 patients [88], and two further retrospective studies with the FIASMA and calcium-channel blocker amlodipine also reported lower mortality rates in patients receiving amlodipine [91,92]. An observational study noted a reduced risk of intubation and death in patients who received a FIASMA within 48 h of hospitalization [93]. A recent lipid metabolic study noted distinct lipid profiles in COVID-19 patient serum depending on disease severity and the increases in several ceramide species belonged to the most discriminant changes [94]. While the pathological consequence of this is still unclear in the context of COVID-19, it has been suggested that increased plasma ceramide levels are a risk factor for cardiovascular events [95]. Thus, the use of FIASMAs may help to mitigate this. 

Other sphingolipids may also be useful in controlling SARS-CoV-2 infections. Sphingosine, for example, was shown to prevent the binding of the viral spike protein to angiotensin-converting enzyme 2 (ACE2) [96]. Ceramidase treatment, which results in the conversion of ceramide to sphingosine, was shown to prevent SARS-CoV-2 spike-mediated entry [85]. Additionally, it may be beneficial in mitigating inflammatory damage, as acid ceramidase inhalation was previously reported to reduce airway inflammation in a cystic fibrosis model [97]. For more information on sphingolipids as potential therapeutic targets for controlling SARS-CoV-2 infection and alleviating COVID-19 symptoms, the reader is referred to recent reviews on this topic [90,98,99,100,101,102]. 

## 3. Viral Gene Expression and Replication 

Once inside the cell, the virus particle needs to reach an appropriate site for genome replication through intracellular trafficking. As mentioned previously, the viral genome also needs to become accessible for cellular enzymes in a process called uncoating, which is often linked to endocytotic entry. Then viral gene expression and replication can occur, and new viral capsids can be assembled.

### 3.1. Viral Replication and Assembly

In these post-entry steps, sphingolipid-metabolizing enzymes are often reported to be important mediators. For example, sphingomyelin turnover and glycolipid synthesis play a role in HSV-1 infection, with inhibition or genetic deficiency for ASM and alterations of glycosphingolipid synthesis interfering substantially with virus reproduction [103,104]. Exogenous ceramide or sphingomyelinase treatment also activates HIV expression, inducing the switch from latent to productive infection [105,106].

Sphingolipid-metabolizing enzymes may affect viral replication through effects on viral transcription factors: IE1 is a major viral transcriptional transactivator of human cytomegalovirus (HCMV) [107]. Machesky and colleagues noted increased sphingosine kinase 1 (SphK1) activity and increases in dihydrosphingosine 1-phosphate (dhS1P) and ceramide levels in response to HCMV infection. The knockdown of SphK1 diminishes the accumulation of IE1, and the pharmacological inhibition of SphK1 has antiviral effects, whereas SphK1 overexpression achieves the reverse [108]. Additionally, another study reported the inhibition of HCMV replication by sphingomyelinase treatment, but enhanced replication in response to short-chain ceramides [109]. 

Rather than affecting viral proteins, sphingolipids may also act on host enzymes. Viruses frequently co-opt the mammalian target of rapamycin (mTOR) pathway, a central regulator of gene expression, translation and metabolic processes, in order to support their own replication [110]. SphK1 and acid ceramidase inhibitors reduce mTORC1 phosphorylation through decreased intracellular S1P levels and thus inhibit MV replication without affecting viral uptake [111]. 

Viruses can remodel intracellular membranes to form replication sites. Zika virus (ZIKV), for example, dysregulates the lipid landscape of infected host cells, particularly with regard to sphingolipids [112]. Ceramide redistributes to the ZIKV replication site and the disruption of sphingolipid biosynthesis blocks ZIKV infection [112]. Ceramide also redistributes to West Nile virus (WNV) replication sites and ceramide production via the de novo and salvage pathways necessary for WNV replication [113]. SM and ceramide transfer protein (CERT) are required for the biosynthesis of double-membrane vesicles that serve as HCV replication sites [114]. In contrast, ceramide does not redistribute into the replication sites of another flavivirus, dengue virus, and the inhibition of ceramide synthase actually enhanced dengue virus production [113], demonstrating that even viruses from the same genus can have different sphingolipid-requirements for replication [115].

Sphingolipids can also regulate intracellular transport, both of incoming virions to their replication site, as well as of new viral products for the assembly of new virions. One example is the ceramide-mediated trafficking of the M glycoprotein of infectious bronchitis virus (IBV) [116]. Another example is the surface display of IAV glycoproteins, which is dependent on sphingomyelin synthase (SMS) and GSL synthesis [117,118]. Sphingolipid-metabolizing enzymes also regulate IAV nuclear export: IAV activates SphK1, which is important for the activation of Ran-binding protein 3 (RanBP3), a co-factor in the nuclear export of the viral ribonucleoprotein complex. Inhibition of SphK1 interferes with nuclear export, reduces the synthesis of viral RNAs and proteins and suppresses virus-induced NFκB activation [119]. SphK1-inhibition also provides protection to IAV-infected mice [120]. Further, the inhibition of de novo ceramide synthesis enhances IAV replication [121], and exogenous ceramide reduces viral titers [121], further suggesting a role of ceramide and its downstream products in IAV replication, protein transport and assembly [121]. 

Finally, viruses may affect sphingolipid metabolism in order to prolong the survival of infected cells, thus winning more time for the replication and assembly of new virions. One example is the activation of SphK1 by respiratory syncytial virus, which prolongs the survival of infected cells [122]. HSV has evolved mechanisms to block apoptosis at multiple metabolic checkpoints, including ceramide-induced apoptosis [123], and HIV-1 *nef* expression results in increased ceramide production in response to TNF-α, yet apoptosis is actually inhibited and proliferation promoted by this, putatively through Nef interfering with AP-1 activation [124,125,126].

### 3.2. Anti-Viral Properties of Ceramide-Metabolism Inhibitors 

A number of compounds are known inhibitors of ceramide metabolism. Myriocin is a potent inhibitor of serine palmitoyltransferase (SPT) [127] and inhibits HCV [128,129] and HBV [130] replication. However, it was later suggested that this effect is due to its structural similarity to sphingosine, rather than its inhibitory effect on SPT [131]. Myriocin treatment also blocks the surface trafficking of influenza proteins [117] and inhibits WNV replication [113]. In contrast, myrocin treatment enhances replication of DENV [113], certain rhinoviruses [132] and enhanced HSV-2 infection [133]. 

Fumonisin B inhibits ceramide synthase [134] and was reported to inhibit IAV replication by disturbing HA trafficking [118] and to block HIV-1 infectivity [135,136]. Similar to myriocin, fumonisin B inhibited WNV replication but enhanced DNV replication [113]. 

D609 (tricyclo-decane-9-yl-xanthogenate) was first described as an inhibitor of phsphatidylcholine-specific phospholipase C [137]. Its inhibition of SMS was identified later [138,139]. The first antiviral reports predate both discoveries and were made in 1984 with HSV-1 and papilloma viruses [140]. Since then, the antiviral properties of D609 were also described for VSV [141], HIV-1 [142], SV40 [143], RSV [144] and rhinovirus [145]. Mechanistically, D609 inhibits viral protein phosphorylation and replication [141,144,146]. It has, however, not been confirmed so far that this is indeed due to the inhibition of SMS. 

Fenretinide, which increases ceramide levels through the stimulation of de novo synthesis and inhibition of SM synthesis [147], was reported to inhibit DENV [148] and ZIKV [149,150] replication by blocking the nuclear import of viral proteins [149]. 

The antiviral effects of ceramide-metabolism modulators are summarized in Table 2 (irrespective of which step in the viral life cycle they inhibit). 

## 4. Virion Release

Once assembly of the new viral capsids is complete, the next step is the *release* from the infected cell. For naked viruses, this occurs through cell lysis. In the case of enveloped viruses, envelopment, a process in which the capsid becomes surrounded by a lipid bilayer, has to happen first. This can occur in a coupled mechanism with capsid assembly, or sequentially, after capsid assembly is completed. Envelopment occurs either at the plasma membrane, resulting in the direct release of the new virions (budding) or at endosomal membranes, followed by exocytosis [8]. 

Not many reports address the role of sphingolipids in lytic viral release. Ceramide is necessary for the lytic phase of adenovirus infection through the regulation of the cellular spliceosome [155]. Of note, adenovirus induces increases in ceramide levels [156] but specifically blocks ceramide-induced apoptosis through the expression of its E1B19K anti-apoptotic protein [155]. 

With regard to budding, neutral sphingomyelinase controls the budding of extracellular vesicles from the plasma membrane [157]. Some viruses seem to exploit this mechanism, as NSM inhibition suppressed ZIKV [153] and WNV release [152]. On the other hand, the membrane composition of HIV-1 and murine leukemia virus differs from that of microvesicles released from their host cell, suggesting a different budding mechanism [158]. 

While the budding of HIV-1 typically occurs at the plasma membrane in productively infected cells, infected macrophages assemble new virions in subcellular, virus-containing compartments, from which they can be released in response to extracellular ATP. Imipramine, a FIASMA, was able to block this release, suggesting a dependency on ASM [151]. 

Another example of sphingolipid-mediated viral release from an intracellular compartment is HCV. HCV particles form through budding into the ER and pass through the Golgi [159]. HCV gene expression downregulates protein kinase D (PKD) activation and thus prevents the inhibition of oxysterol-binding protein (OSBP) and CERT [160]. OSBP and CERT form a membrane contact site between the ER and the trans Golgi network (TGN) and transfer ceramide, cholesterols and oxysterols to the TGN, resulting in the microdomain formation required for HCV secretion [40,160,161,162]. Treatment with CERT-inhibitor (1R, 3R)-*N*-(3-hydroxy-1-hydroxymethyl-3-phenylpropyl)dodecanamide (HPA-12) inhibited HCV replication [154]. This PKD pathway is also important for HSV-1 egress. In HSV-1 infections, however, CERT also appears to modulate viral egress independently of its lipid transfer properties at a previous stage than the release of virions from the cell [163]. 

The lipid composition of new virions is not solely a byproduct of the release process but can also determine infectivity. HCV infectivity, for instance, is abolished almost completely by the depletion of cholesterol or the hydrolysis of virion-associated sphingomyelin, and a significant portion of HCV structural proteins partition into rafts [40]. This suggests that membrane microdomains on virion membranes are similarly important for the interaction of viral glycoproteins with their cellular receptors in viral entry as microdomains on the cell-to-be-infected. In line with this, the glycoproteins of IAV [164], HIV [165], MV [166,167], EBOV and Marburg virus [168] have also been associated with membrane rafts and alterations of HIV-1 lipid composition reduced infectivity [136,169]. 

Stopping viral spread by preventing the release of new virions is a potential therapeutic strategy. In addition to targeting sphingomyelinases in order to prevent the budding of virions, sialidase inhibition was described as a means to stop IAV spread: sialidase cleaves the link between newly formed influenza virions and the cell surface, liberating the virions from the cell. Synthetic ganglioside analogs inhibit viral sialidase and thus viral dissemination [170]. 

## 5. Viral-Induced Apoptosis and Morbidity 

The death of infected cells can be a double-edged sword for both the host and the virus. Generally, the death of an infected cell in response to virus recognition is considered beneficial for the host, since it destroys the intracellular niche of the pathogen [171]. As discussed earlier, many viruses have developed strategies that block cell-death signals in order to avoid or delay the demise of the host cell, thus gaining more time for replication. This can even still occur during latent infection: latency associated transcript (LAT) is the only HSV-1 gene that is abundantly transcribed during latency, and it inhibits apoptosis, including ceramide-induced apoptosis [172]. 

Despite usually curbing viral replication, viral-induced cell death can also be detrimental for the host depending on the context: apoptosis may promote the release of new virions from infected cells, enhancing virus dissemination. It may contribute to viral disease by causing tissue injury and it may blunt the immune response [173]. Examples of how viral-induced apoptosis contributes to morbidity are encephalomyelitis, due to Sindbis virus-induced neuronal cell death [174,175]; erythroid aplastic crisis, due to B19V infection of erythroid progenitor cells [176]; and dementia, due to HIV-1-induced neuronal apoptosis [177,178,179]. In all of these cases, the viral-induced apoptosis involves sphingomyelinase activation and ceramide generation. 

Viral infections have also been suggested to play a role in the development of autoimmune diseases through a process called “molecular mimicry”: Antibodies generated against viruses, e.g., Theiler’s murine encephalomyelitis virus, SFV, IAV, MV and rubella virus also bind various lipid-like structures, including the myelin component galactocerebroside, which may contribute to myelin destruction [180,181,182,183,184]. On the plus side, cross-reactive immunity arising from a common host cell-membrane-derived glycolipid component present in the viral envelopes of different viruses may protect against other viral infections. For example, SFV infection reduces morbidity from a subsequent Langat virus infection [185].

## 6. Viral Immune Evasion and Immune Modulation

### 6.1. Viral Immune Evasion

Viruses have developed different strategies to escape immune surveillance. Studies on HIV-1 led to the “Trojan endosome hypothesis”, postulating that retroviruses can exploit intercellular vesicle traffic for both biogenesis of retroviral particles as well as for receptor-independent infection [186]. Since the discovery of this low-efficiency but mechanistically important mode of infection for HIV-1, exosomes produced by hepatitis B- and E-virus-infected cells have also been shown to contain infectious particles of the respective virus [187,188]. Importantly, packaging in exosomes provides protection from antibody neutralization [187,188]. Since sphingomyelinases control the biogenesis of extracellular vesicles, this evasion process is linked to sphingolipid-metabolism. 

Another example is MV-induced immunosuppression, which occurs despite efficient virus-specific immune activation. MV causes immunosuppression mainly through suppression of T cells: ceramide generation by ASM and NSM upon contact with MV contributes to actin cytoskeletal paralysis, resulting in the loss of T cell polarization, adhesion and motility [189,190,191]. 

### 6.2. Invariant Natural Killer T Cells in Viral Infections

Invariant natural killer T cells (iNKT) couple the rapid activation kinetics of innate immune cells with the diverse functions of adaptive T cells [192]. Through their rapid and broad effector functions, including the production of many cytokines and chemokines, perforin/granzyme release, Fas/FasL-mediated cytotoxicity, activation of other immune cells and enhancement of CD4^+^ and CD8^+^ antigen-specific responses, iNKT cells contribute to viral clearance [193]. For example, ceramide synthase 2 null mice are susceptible to lymphocytic choriomeningitis virus (LCMV) due to reduced iNKT cell numbers [194]. In chronic HIV infection, NKT cells are depleted, and an early loss of NKT cells is associated with subsequent immune destruction during HIV infection [195]. 

Rather than a peptide antigen presented on an MHC molecule, iNKT cells express a highly restricted T cell receptor that recognizes alpha-galactosylceramide (α-GalCer) and a few other glycolipid antigens presented by CD1d [196]. As viral genomes do not generate lipid molecules, it is unclear how virus-infected cells activate iNKT cells. One way that has been reported is through the interaction of iNKT cells with dendritic cells, which upregulate CD1d expression in response to viral danger signals [197]. Similarly, sphingolipid pathways were reported to be altered by the HIV-1 infection of dendritic cells, upregulating α-GalCer expression [198]. Cytokine-mediated activation of iNKT cells may also occur [199]. 

Viruses have developed evasion strategies to avoid the activation of iNKT cells. For instance, HIV-1 Nef and Vpu interfere with CD1d surface expression [198]. Similarly, LCVM infection also causes a reduction in CD1d expression, whereas vaccinia virus and VSV alter the intracellular trafficking of CD1d molecules, and HSV-1 alters CD1d recycling [200]. Additionally, direct contact with HSV-1-infected cells alters T cell receptor signaling in the iNKT cells downstream of ZAP70 [201]. 

### 6.3. Potential Clinical Applications of α-GalCer

iNKT activation by α-GalCer promotes the development of long-term protective immunity by increasing the fitness of central memory CD8^+^ T cells [202]. This rationale underlies a plethora of studies investigating the use of α-GalCer as a vaccine adjuvant. So far, α-GalCer has been tested as an adjuvant for human papillomavirus (HPV) [203], IAV [204,205,206,207,208,209,210,211,212,213], HSV-2 [214,215], HIV-1 [216,217] and human metapneumovirus [218] vaccines. 

α-GalCer is also being investigated as a so-called “B cell vaccine”: α-GalCer-loaded, antigen-expressing B cells could be an alternative to dendritic cells in immunotherapy by stimulating antigen-specific T cells and B cells [219,220]. 

In a transgenic mouse model of chronic hepatitis B infection, α-GalCer could abolish hepatitis B virus (HBV) replication [221] and overcome tolerance to HBV antigens [222]. α-GalCer has already been tested as a monotherapy for interferon-refractory chronic hepatitis C. While treatment was safe and showed moderate immunomodulatory effects, the administered doses had no significant effect on HCV RNA levels [221]. 

The activation of iNKT cells with α-GalCer during influenza infection ameliorated morbidity in a mouse model through enhanced early innate immune response and reduced viral titers [223]. Furthermore, it could limit bacterial superinfection post influenza [224]. In coxsackievirus b3-infected mice, α-GalCer treatment reduced myocarditis but increased liver pathogenesis [225,226]. In encephalomyocarditis virus (EMCV-D)-infected mice, α-GalCer protected against encephalitis, myocarditis and diabetes [227].

α-GalCer also elicits antiviral effect against HBV and HCV through induction of the 2′,5′ oligoadenylate synthase gene family and the secretion of beta interferon [228]. 

## 7. Conclusions

**Figure 3:**
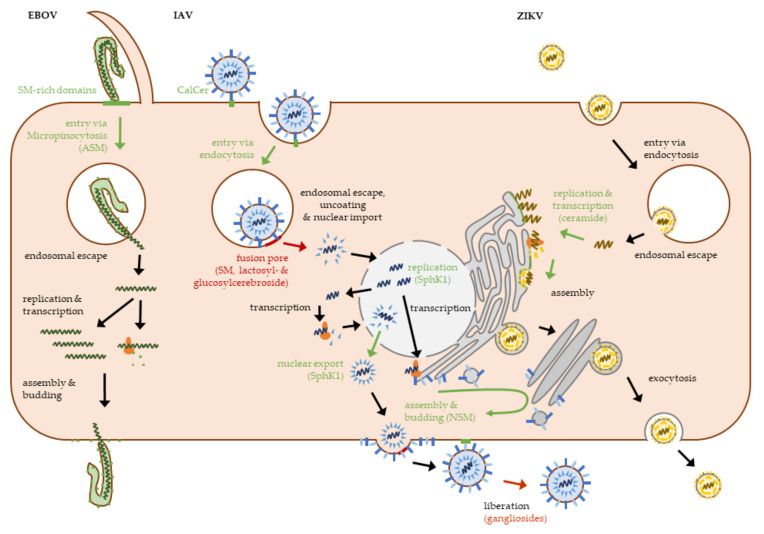
Role of sphingolipids in ebola-, influenza- and Zika virus replication. The life cycles of ebola virus (EBOV), influenza A virus (IAV) and Zika virus (ZIKV) show some of the diversity of viral replication and summarize key steps in which sphingolipids play a role in the viral life cycle. Supporting roles, i.e., the virus using sphingolipid-domains for cell entry or the formation of replication sites, are highlighted in green. Inhibitory roles are marked in red. ASM: acid sphingomyelinase, NSM: natural sphingomyelinase, SM: sphingomyelin, SphK1: sphingosine 1-kinase.

Ceramide and its related molecules contribute to all stages of the viral life cycle. Figure 3 summarizes this for three different viruses, which also serve as an example for the diversity of viral replication (Figure 3). 

Targeting sphingolipid-metabolizing enzymes offers interesting new opportunities for antiviral therapies. Inhibitors of ceramide metabolism like fenretinide, PDMP, myriocin, Fumosin B, 12-HPA and FIASMAs have been reported to have antiviral properties against a multitude of different viruses (Table 2). Future work should focus on further defining the involvement of sphingolipids in viral entry, replication and release with the hope of identifying new antiviral therapeutic targets. Of particular current interest in light of the SARS-CoV-2/COVID-19 pandemic is the repurposing of FIASMAs for the inhibition of SARS-CoV-2 entry [83,84,88,89,90,91,92,93]. The long-standing clinical experience with these drugs and their favorable pharmacological properties, including good absorption, distribution, metabolism and excretion, lack of habituation, reversible inhibition and lack of rebound effects [229] make them ideal candidates for a swift indication expansion to manage SARS-COV-2 infection/COVID-19. They may also provide an economic treatment option, particularly in countries that struggle with financing the vaccination program and where SARS-COV-2 will likely become endemic [230]. 

Beyond SARS-CoV-2, sphingomyelinase inhibitors have the potential to inhibit viral entry and dissemination in a broader range of viruses (summarized in Table 2 and Table 3). Additionally, α-GalCer harbors great potential as an adjuvant and immune modulator in viral infections [203,204,205,206,207,208,209,210,211,212,213,214,215,216,217,218,219,220,221,223,224,225,226,227]. Future studies will have to carefully consider the effect of sphingolipid modulation for the respective virus studied, however, as even the same agent can have opposing biological effects for different viruses [55,64].

## Figures and Tables

**Table 1:** Viruses using sphingolipids for cell attachment.

Virus	Receptor/Pathway	Reference
ebola virus (EBOV)	SM-rich regions	[39]
hepatitis C virus (HCV)	viral sphingomyelin required for internalization	[40]
human immunodeficiency virus type I (HIV-1)	GalCer, Gb3, GM3, SMS2	[13,15,16,17,18,19,20,21,22,23,41,42,43]
human parvovirus B19 (B19V)	Gb4Cer	[37,38]
influenza A virus (IAV)	CalCer	[26,27]
measles virus (MV)	ASM-dependent CD150 surface localization	[44,45]
murine norovirus	serine palmitoyltransferase-dependent conformation of CD300lf	[36]
norovirus GII.4	CalCer	[28]
rhinovirus	ceramide-enriched platforms	[30,31,32]
rotavirus	GA1, GA2, pentaosylceramides	[11,12]
rubella virus	SM and cholesterol	[29]
severe acute respiratory syndrome coronavirus type 2 (SARS-CoV-2)	ACE2 in lipid rafts	[34]
simian virus 40 (SV40)	GM1, N-glycolyl GM1	[46,47]

**Table 2:** Antiviral effects of ceramide-metabolism modulators.

Substance	Target Molecule (effect)	Virus	Reference
D609	SMS (−)	HIV-1 Rhinovirus RSV SV40 VSV	[142] [145] [144] [143] [141]
FIASMAs	ASM (−)	Adenovirus EBOV HIV-1 IAV JEV MV Norovirus Rhinovirus SARS-CoV-2	[57] [39] [151] [84] [86] [44] [58,69] [30] [83,84,85,92,93]
Fenretinide	SPT (+) dihydroceramide desaturase (−)	DEN HIV-1 ZIKV	[148] [55] [149,150]
Fumosin B	CerS (−)	IAV WNV	[118] [113]
GW4869	NSM (−)	WNV ZIKV	[152] [112,153]
12-HPA	CERT (−)	HCV	[154]
Myriocin	SPT (−)	HBV HCV IAV WNV	[130] [128,129] [117,118] [113]
SKI-II	SphK (−)	HCMV IAV MV	[108] [120] [111]

**Table 3:** Effects of sphingomyelinase inhibitors on different viruses.

Virus	Inhibitor (Target Molecule)	Effect	Reference
adenovirus	Fluoxetine, Amitriptyline (ASM)	block endosomal escape	[57]
EBOV	Imipramine, Desipramine (ASM)	prevent entry	[39]
HIV-1	Imipramine (ASM)	decreases release	[151]
	GW4869 (NSM)	protects from neuronal cell death	[231]
IAV	Fluoxetine, Amiodarone, Imipramine (ASM)	reduce viral titers	[84]
	Desipramine (ASM))	no effect	[64]
JEV	Amitriptyline (ASM)	reduces infection	[86]
MV	GW4869 (NSM), Amitriptyline (ASM)	mitigate T cell suppression	[191]
	Amitriptyline (ASM)	inhibits uptake	[44]
norovirus	AY9944, Fluoxetine, Desipramine, Chlorpromazine, Amitriptyline (ASM)	reduce viral titers	[58,69]
	Desipramine	blocks endosomal escape	[69]
	GW4869 (NSM)	no effect	[58]
rhinovirus	Amitriptyline, Imipramine (ASM)	inhibit uptake	[30]
SARS-CoV-2	Amitriptyline (ASM), Ambroxol (ASM)	prevent entry	[83,85]
	Fluoxetine, Amiodarone, Imipramine (ASM)	reduce viral titers	[84]
	Amlodipine (ASM)	reduces mortality	[88,92]
	Fluoxetine (ASM)	lowers risk of intubation and reduces mortality	[93]
WNV	GW4869 (NSM)	decreases release	[152]
ZIKV	GW4869 (NSM)	decreases production and shedding	[112,153]

## Abbreviations

Viruses	
B19V	human parvovirus B19
EBOV	ebola virus
EMCV-D	encephalomyocarditis virus
HBV	hepatitis B virus
HCMV	human cytomegalovirus
HCV	hepatitis C virus
HIV-1	human immunodeficiency virus type 1
HPV	human papillomavirus
HSV-1	herpes simplex virus 1
HSV-2	herpes simplex virus 2
IAV	influenza A virus
IBV	infectious bronchitis virus
JEV	Japanese encephalitis virus
LCMV	lymphocytic choriomeningitis virus
MV	measles virus
SARS-CoV	severe acute respiratory syndrome corona virus
SFV	Semliki Forest virus
SV40	simian virus 40
VSV	vesicular stomatitis virus
WNV	West Nile virus
ZIKV	Zika virus
Sphingolipids and sphingolipid-metabolizing enzymes	
α-CalCer	α-galactosylceramide
ASM	acid sphingomyelinase
CERT	ceramide transport protein
dhS1P	dihydrosphingosine 1-phosphate
FIASMA	functional inhibitor of acid sphingomyelinase
GalCer	galactosylceramide
Gb3	globotriaosylceramide
Gb4Cer	globoside/globotetraosylceramide
GlcCerS	glucosylceramide synthase
GM3-NeuAc	N-acetylneuraminyllactosylceramide
GSL	glycosphingolipids
NSM	neutral sphingomyelinase
SM	sphingomyelin
SMS2	sphingomyelin synthase 2
SphK1	sphingosine kinase 1
SPT	serine palmitoyltransferase
Other	
ACE2	angiotensin converting enzyme 2
COVID-19	corona virus disease 2019
DC-SIGN	dendritic cell-specific intracellular adhesion molecule-3-grabbing non-integrin
HA	hemagluttinin
iNKT	invariant natural killer T cell
mTOR	mammalian target of rapamycin
OSBP	oxysterol-binding protein
PKD	protein kinase D
RanBP3	Ran-binding protein 3
TGN	trans golgi network

## References

[1] Narimatsu S., Soeda S., Tanaka T., Kishimoto Y. (1986). Solubilization and partial characterization of fatty acyl-CoA:sphingosine acyltransferase (ceramide synthetase) from rat liver and brain. Biochim. Biophys. Acta.

[2] Gault C.R., Obeid L.M., Hannun Y.A. (2010). An overview of sphingolipid metabolism: From synthesis to breakdown. Adv. Exp. Med. Biol..

[3] Schneider P.B., Kennedy E.P. (1967). Sphingomyelinase in normal human spleens and in spleens from subjects with Niemann-Pick disease. J. Lipid. Res..

[4] Beckmann N., Gulbins E., Becker K.A., Carpinteiro A., Choi S. (2017). Sphingomyelinase, Acidic. Encyclopedia of Signaling Molecules.

[5] Grassme H., Jekle A., Riehle A., Schwarz H., Berger J., Sandhoff K., Kolesnick R., Gulbins E. (2001). CD95 signaling via ceramide-rich membrane rafts. J. Biol. Chem..

[6] Beckmann N., Sharma D., Gulbins E., Becker K.A., Edelmann B. (2014). Inhibition of acid sphingomyelinase by tricyclic antidepressants and analogons. Front. Physiol..

[7] Draeger A., Babiychuk E.B. (2013). Ceramide in plasma membrane repair. Handb. Exp. Pharmacol..

[8] Ryu W.-S., Ryu W.-S. (2017). Chapter 3—Virus Life Cycle. Molecular Virology of Human Pathogenic Viruses.

[9] Chen C.S., Rosenwald A.G., Pagano R.E. (1995). Ceramide as a modulator of endocytosis. J. Biol. Chem..

[10] Volpert G., Ben-Dor S., Tarcic O., Duan J., Saada A., Merrill A.H., Pewzner-Jung Y., Futerman A.H. (2017). Oxidative stress elicited by modifying the ceramide acyl chain length reduces the rate of clathrin-mediated endocytosis. J. Cell Sci..

[11] Srnka C.A., Tiemeyer M., Gilbert J.H., Moreland M., Schweingruber H., de Lappe B.W., James P.G., Gant T., Willoughby R.E., Yolken R.H. (1992). Cell surface ligands for rotavirus: Mouse intestinal glycolipids and synthetic carbohydrate analogs. Virology.

[12] Martínez M.A., López S., Arias C.F., Isa P. (2013). Gangliosides Have a Functional Role during Rotavirus Cell Entry. J. Virol..

[13] Fantini J., Hammache D., Piéroni G., Yahi N. (2000). Role of glycosphingolipid microdomains in CD4-dependent HIV-1 fusion. Glycoconj. J..

[14] Chen B. (2019). Molecular Mechanism of HIV-1 Entry. Trends Microbiol..

[15] Dennison S.M., Anasti K.M., Jaeger F.H., Stewart S.M., Pollara J., Liu P., Kunz E.L., Zhang R., Vandergrift N., Permar S. (2014). Vaccine-Induced HIV-1 Envelope gp120 Constant Region 1-Specific Antibodies Expose a CD4-Inducible Epitope and Block the Interaction of HIV-1 gp140 with Galactosylceramide. J. Virol..

[16] Magérus-Chatinet A., Yu H., Garcia S., Ducloux E., Terris B., Bomsel M. (2007). Galactosyl ceramide expressed on dendritic cells can mediate HIV-1 transfer from monocyte derived dendritic cells to autologous T cells. Virology.

[17] Alfsen A., Bomsel M. (2002). HIV-1 gp41 Envelope Residues 650–685 Exposed on Native Virus Act as a Lectin to Bind Epithelial Cell Galactosyl Ceramide. J. Biol. Chem..

[18] Cook D.G., Fantini J., Spitalnik S.L., Gonzalez-Scarano F. (1994). Binding of Human Immunodeficiency Virus Type I (HIV-1) Gp120 to Galactosylceramide (GalCer): Relationship to the V3 Loop. Virology.

[19] Yahi N., Baghdiguian S., Moreau H., Fantini J. (1992). Galactosyl ceramide (or a closely related molecule) is the receptor for human immunodeficiency virus type 1 on human colon epithelial HT29 cells. J. Virol..

[20] Harouse J., Bhat S., Spitalnik S., Laughlin M., Stefano K., Silberberg D., Gonzalez-Scarano F. (1991). Inhibition of entry of HIV-1 in neural cell lines by antibodies against galactosyl ceramide. Science.

[21] Puri A., Hug P., Jernigan K., Barchi J., Kim H.-Y., Hamilton J., Wiels J., Murray G.J., Brady R.O., Blumenthal R. (1998). The neutral glycosphingolipid globotriaosylceramide promotes fusion mediated by a CD4-dependent CXCR4-utilizing HIV type 1 envelope glycoprotein. Proc. Natl. Acad. Sci. USA.

[22] Puri A., Hug P., Jernigan K., Rose P., Blumenthal R. (1999). Role of Glycosphingolipids in HIV-1 Entry: Requirement of Globotriosylceramide (Gb3) in CD4/CXCR4-dependent Fusion. Biosci. Rep..

[23] Hammache D., Yahi N., Maresca M., Piéroni G., Fantini J. (1999). Human Erythrocyte Glycosphingolipids as Alternative Cofactors for Human Immunodeficiency Virus Type 1 (HIV-1) Entry: Evidence for CD4-Induced Interactions between HIV-1 gp120 and Reconstituted Membrane Microdomains of Glycosphingolipids (Gb3 and GM3). J. Virol..

[24] Popik W., Alce T.M., Au W.C. (2002). Human immunodeficiency virus type 1 uses lipid raft-colocalized CD4 and chemokine receptors for productive entry into CD4(+) T cells. J. Virol..

[25] Kamiyama H., Yoshii H., Tanaka Y., Sato H., Yamamoto N., Kubo Y. (2009). Raft localization of CXCR4 is primarily required for X4-tropic human immunodeficiency virus type 1 infection. Virology.

[26] Suzuki T., Sometani A., Yamazaki Y., Horiike G., Mizutani Y., Masuda H., Yamada M., Tahara H., Xu G., Miyamoto D. (1996). Sulphatide binds to human and animal influenza A viruses, and inhibits the viral infection. Biochem. J..

[27] Suzuki Y., Matsunaga M., Matsumoto M. (1985). N-Acetylneuraminyllactosylceramide, GM3-NeuAc, a new influenza A virus receptor which mediates the adsorption-fusion process of viral infection. Binding specificity of influenza virus A/Aichi/2/68 (H3N2) to membrane-associated GM3 with different molecular species of sialic acid. J. Biol. Chem..

[28] Bally M., Rydell G.E., Zahn R., Nasir W., Eggeling C., Breimer M.E., Svensson L., Höök F., Larson G. (2012). Norovirus GII.4 Virus-like Particles Recognize Galactosylceramides in Domains of Planar Supported Lipid Bilayers. Angew. Chem. Int. Ed..

[29] Otsuki N., Sakata M., Saito K., Okamoto K., Mori Y., Hanada K., Takeda M. (2018). Both Sphingomyelin and Cholesterol in the Host Cell Membrane Are Essential for Rubella Virus Entry. J. Virol..

[30] Grassmé H., Riehle A., Wilker B., Gulbins E. (2005). Rhinoviruses Infect Human Epithelial Cells via Ceramide-enriched Membrane Platforms. J. Biol. Chem..

[31] Dreschers S., Franz P., Dumitru C.A., Wilker B., Jahnke K., Gulbins E. (2007). Infections with Human Rhinovirus Induce the Formation of Distinct Functional Membrane Domains. Cell. Physiol. Biochem..

[32] Bentley J.K., Newcomb D.C., Goldsmith A.M., Jia Y., Sajjan U.S., Hershenson M.B. (2007). Rhinovirus Activates Interleukin-8 Expression via a Src/p110β Phosphatidylinositol 3-Kinase/Akt Pathway in Human Airway Epithelial Cells. J. Virol..

[33] Hoffmann M., Kleine-Weber H., Schroeder S., Krüger N., Herrler T., Erichsen S., Schiergens T.S., Herrler G., Wu N.-H., Nitsche A. (2020). SARS-CoV-2 Cell Entry Depends on ACE2 and TMPRSS2 and Is Blocked by a Clinically Proven Protease Inhibitor. Cell.

[34] Lu Y., Liu D.X., Tam J.P. (2008). Lipid rafts are involved in SARS-CoV entry into Vero E6 cells. Biochem. Biophys. Res. Commun..

[35] Orchard R.C., Wilen C.B., Doench J.G., Baldridge M.T., McCune B.T., Lee Y.C., Lee S., Pruett-Miller S.M., Nelson C.A., Fremont D.H. (2016). Discovery of a proteinaceous cellular receptor for a norovirus. Science.

[36] Orchard R.C., Wilen C.B., Virgin H.W. (2018). Sphingolipid biosynthesis induces a conformational change in the murine norovirus receptor and facilitates viral infection. Nat. Microbiol..

[37] Quattrocchi S., Ruprecht N., Bönsch C., Bieli S., Zürcher C., Boller K., Kempf C., Ros C. (2012). Characterization of the Early Steps of Human Parvovirus B19 Infection. J. Virol..

[38] Bönsch C., Zuercher C., Lieby P., Kempf C., Ros C. (2010). The Globoside Receptor Triggers Structural Changes in the B19 Virus Capsid That Facilitate Virus Internalization. J. Virol..

[39] Miller M.E., Adhikary S., Kolokoltsov A.A., Davey R.A. (2012). Ebolavirus Requires Acid Sphingomyelinase Activity and Plasma Membrane Sphingomyelin for Infection. J. Virol..

[40] Aizaki H., Morikawa K., Fukasawa M., Hara H., Inoue Y., Tani H., Saito K., Nishijima M., Hanada K., Matsuura Y. (2008). Critical Role of Virion-Associated Cholesterol and Sphingolipid in Hepatitis C Virus Infection. J. Virol..

[41] Hug P., Lin H.M., Korte T., Xiao X., Dimitrov D.S., Wang J.M., Puri A., Blumenthal R. (2000). Glycosphingolipids promote entry of a broad range of human immunodeficiency virus type 1 isolates into cell lines expressing CD4, CXCR4, and/or CCR5. J. Virol.

[42] Puri A., Rawat S.S., Lin H.M., Finnegan C.M., Mikovits J., Ruscetti F.W., Blumenthal R. (2004). An inhibitor of glycosphingolipid metabolism blocks HIV-1 infection of primary T-cells. Aids.

[43] Hayashi Y., Nemoto-Sasaki Y., Tanikawa T., Oka S., Tsuchiya K., Zama K., Mitsutake S., Sugiura T., Yamashita A. (2014). Sphingomyelin Synthase 2, but Not Sphingomyelin Synthase 1, Is Involved in HIV-1 Envelope-mediated Membrane Fusion. J. Biol. Chem..

[44] Avota E., Gulbins E., Schneider-Schaulies S. (2011). DC-SIGN Mediated Sphingomyelinase-Activation and Ceramide Generation Is Essential for Enhancement of Viral Uptake in Dendritic Cells. PLoS Pathog..

[45] Avota E., Koethe S., Schneider-Schaulies S. (2013). Membrane dynamics and interactions in measles virus dendritic cell infections. Cell. Microbiol..

[46] Tsai B., Gilbert J.M., Stehle T., Lencer W., Benjamin T.L., Rapoport T.A. (2003). Gangliosides are receptors for murine polyoma virus and SV40. EMBO J..

[47] Campanero-Rhodes M.A., Smith A., Chai W., Sonnino S., Mauri L., Childs R.A., Zhang Y., Ewers H., Helenius A., Imberty A. (2007). N-glycolyl GM1 ganglioside as a receptor for simian virus 40. J. Virol..

[48] Greber U.F., Singh I., Helenius A. (1994). Mechanisms of virus uncoating. Trends Microbiol..

[49] Ou X., Liu Y., Lei X., Li P., Mi D., Ren L., Guo L., Guo R., Chen T., Hu J. (2020). Characterization of spike glycoprotein of SARS-CoV-2 on virus entry and its immune cross-reactivity with SARS-CoV. Nat. Commun..

[50] Shirato K., Kanou K., Kawase M., Matsuyama S. (2017). Clinical Isolates of Human Coronavirus 229E Bypass the Endosome for Cell Entry. J. Virol..

[51] Sun W., He S., Martínez-Romero C., Kouznetsova J., Tawa G., Xu M., Shinn P., Fisher E.G., Long Y., Motabar O. (2017). Synergistic drug combination effectively blocks Ebola virus infection. Antivir. Res..

[52] Simmons G., Reeves J.D., Grogan C.C., Vandenberghe L.H., Baribaud F., Whitbeck J.C., Burke E., Buchmeier M.J., Soilleux E.J., Riley J.L. (2003). DC-SIGN and DC-SIGNR bind ebola glycoproteins and enhance infection of macrophages and endothelial cells. Virology.

[53] Voisset C., Lavie M., Helle F., De Beeck A.O., Bilheu A., Bertrand-Michel J., Tercé F., Cocquerel L., Wychowski C., Vu-Dac N. (2008). Ceramide enrichment of the plasma membrane induces CD81 internalization and inhibits hepatitis C virus entry. Cell. Microbiol..

[54] Finnegan C.M., Rawat S.S., Puri A., Wang J.M., Ruscetti F.W., Blumenthal R. (2004). Ceramide, a target for antiretroviral therapy. Proc. Natl. Acad. Sci. USA.

[55] Finnegan C.M., Blumenthal R. (2006). Fenretinide inhibits HIV infection by promoting viral endocytosis. Antivir. Res..

[56] Gobeil L.-A., Lodge R., Tremblay M.J. (2012). Differential HIV-1 Endocytosis and Susceptibility to Virus Infection in Human Macrophages Correlate with Cell Activation Status. J. Virol..

[57] Luisoni S., Suomalainen M., Boucke K., Tanner L.B., Wenk M.R., Guan X.L., Grzybek M., Coskun Ü., Greber U.F. (2015). Co-option of Membrane Wounding Enables Virus Penetration into Cells. Cell Host Microbe.

[58] Murakami K., Tenge V.R., Karandikar U.C., Lin S.-C., Ramani S., Ettayebi K., Crawford S.E., Zeng X.-L., Neill F.H., Ayyar B.V. (2020). Bile acids and ceramide overcome the entry restriction for GII.3 human norovirus replication in human intestinal enteroids. Proc. Natl. Acad. Sci. USA.

[59] Peters S., Schlegel J., Becam J., Avota E., Sauer M., Schubert-Unkmeir A. (2019). Neisseria meningitidis Type IV Pili Trigger Ca(2+)-Dependent Lysosomal Trafficking of the Acid Sphingomyelinase To Enhance Surface Ceramide Levels. Infect. Immun..

[60] Simonis A., Hebling S., Gulbins E., Schneider-Schaulies S., Schubert-Unkmeir A. (2014). Differential Activation of Acid Sphingomyelinase and Ceramide Release Determines Invasiveness of Neisseria meningitidis into Brain Endothelial Cells. PLoS Pathog..

[61] Gaspar E.B., Mortara R.A., Andrade L.O., da Silva C.V. (2009). Lysosomal exocytosis: An important event during invasion of lamp deficient cells by extracellular amastigotes of Trypanosoma cruzi. Biochem. Biophys. Res. Commun..

[62] Luo M. (2012). Influenza virus entry. Adv. Exp. Med. Biol..

[63] Razinkov V.I., Cohen F.S. (2000). Sterols and Sphingolipids Strongly Affect the Growth of Fusion Pores Induced by the Hemagglutinin of Influenza Virus. Biochemistry.

[64] Audi A., Soudani N., Dbaibo G., Zaraket H. (2020). Depletion of Host and Viral Sphingomyelin Impairs Influenza Virus Infection. Front. Microbiol..

[65] Erdreich-Epstein A., Tran L.B., Bowman N.N., Wang H., Cabot M.C., Durden D.L., Vlckova J., Reynolds C.P., Stins M.F., Groshen S. (2002). Ceramide signaling in fenretinide-induced endothelial cell apoptosis. J. Biol. Chem..

[66] Wilschut J., Corver J., Nieva J.L., Bron R., Moesby L., Reddy K.C., Bittman R. (1995). Fusion of Semliki Forest virus with cholesterol-containing liposomes at low pH: A specific requirement for sphingolipids. Mol. Membr. Biol..

[67] Nieva J.L., Bron R., Corver J., Wilschut J. (1994). Membrane fusion of Semliki Forest virus requires sphingolipids in the target membrane. EMBO J..

[68] Corver J., Moesby L., Erukulla R.K., Reddy K.C., Bittman R., Wilschut J. (1995). Sphingolipid-dependent fusion of Semliki Forest virus with cholesterol-containing liposomes requires both the 3-hydroxyl group and the double bond of the sphingolipid backbone. J. Virol..

[69] Shivanna V., Kim Y., Chang K.-O. (2015). Ceramide formation mediated by acid sphingomyelinase facilitates endosomal escape of caliciviruses. Virology.

[70] Heinrich M., Wickel M., Winoto-Morbach S., Schneider-Brachert W., Weber T., Brunner J., Saftig P., Peters C., Krönke M., Schütze S. (2000). Ceramide as an activator lipid of cathepsin D. Adv. Exp. Med. Biol..

[71] Shivanna V., Kim Y., Chang K.-O. (2014). Endosomal acidification and cathepsin L activity is required for calicivirus replication. Virology.

[72] Le Blanc I., Luyet P.P., Pons V., Ferguson C., Emans N., Petiot A., Mayran N., Demaurex N., Fauré J., Sadoul R. (2005). Endosome-to-cytosol transport of viral nucleocapsids. Nat. Cell Biol..

[73] Nour A.M., Li Y., Wolenski J., Modis Y. (2013). Viral membrane fusion and nucleocapsid delivery into the cytoplasm are distinct events in some flaviviruses. PLoS Pathog..

[74] Lang J., Bohn P., Bhat H., Jastrow H., Walkenfort B., Cansiz F., Fink J., Bauer M., Olszewski D., Ramos-Nascimento A. (2020). Acid ceramidase of macrophages traps herpes simplex virus in multivesicular bodies and protects from severe disease. Nat. Commun..

[75] Guerrero C.A., Zárate S., Corkidi G., López S., Arias C.F. (2000). Biochemical characterization of rotavirus receptors in MA104 cells. J. Virol..

[76] Han Y., Ventura C.L., Black K.P., Cummins J.E., Hall S.D., Jackson S. (2000). Productive human immunodeficiency virus-1 infection of epithelial cell lines of salivary gland origin. Oral Microbiol. Immunol..

[77] Garg H., Francella N., Tony K.A., Augustine L.A., Barchi J.J., Fantini J., Puri A., Mootoo D.R., Blumenthal R. (2008). Glycoside analogs of β-galactosylceramide, a novel class of small molecule antiviral agents that inhibit HIV-1 entry. Antivir. Res..

[78] Fantini J., Hammache D., Delézay O., Yahi N., André-Barrès C., Rico-Lattes I., Lattes A. (1997). Synthetic Soluble Analogs of Galactosylceramide (GalCer) Bind to the V3 Domain of HIV-1 gp120 and Inhibit HIV-1-induced Fusion and Entry. J. Biol. Chem..

[79] Blanzat M., Turrin C.-O., Aubertin A.-M., Couturier-Vidal C., Caminade A.-M., Majoral J.-P., Rico-Lattes I., Lattes A. (2005). Dendritic Catanionic Assemblies: In vitro Anti-HIV Activity of Phosphorus-Containing Dendrimers Bearing Galβ1cer Analogues. ChemBioChem.

[80] Fantini J., Hammache D., Delézay O., Piéroni G., Tamalet C., Yahi N. (1998). Sulfatide Inhibits HIV-1 Entry into CD4−/CXCR4+Cells. Virology.

[81] Lund N., Branch D.R., Mylvaganam M., Chark D., Ma X.-Z., Sakac D., Binnington B., Fantini J., Puri A., Blumenthal R. (2006). A novel soluble mimic of the glycolipid, globotriaosyl ceramide inhibits HIV infection. AIDS.

[82] Perino J., Foo C.H., Spehner D., Cohen G.H., Eisenberg R.J., Crance J.-M., Favier A.-L. (2011). Role of sulfatide in vaccinia virus infection. Biol. Cell.

[83] Carpinteiro A., Edwards M.J., Hoffmann M., Kochs G., Gripp B., Weigang S., Adams C., Carpinteiro E., Gulbins A., Keitsch S. (2020). Pharmacological Inhibition of Acid Sphingomyelinase Prevents Uptake of SARS-CoV-2 by Epithelial Cells. Cell Rep. Med..

[84] Schloer S., Brunotte L., Goretzko J., Mecate-Zambrano A., Korthals N., Gerke V., Ludwig S., Rescher U. (2020). Targeting the endolysosomal host-SARS-CoV-2 interface by clinically licensed functional inhibitors of acid sphingomyelinase (FIASMA) including the antidepressant fluoxetine. Emerg. Microbes Infect..

[85] Carpinteiro A., Gripp B., Hoffmann M., Pöhlmann S., Hoertel N., Edwards M.J., Kamler M., Kornhuber J., Becker K.A., Gulbins E. (2021). Inhibition of acid sphingomyelinase by ambroxol prevents SARS-CoV-2 entry into epithelial cells. J. Biol. Chem..

[86] Tani H., Shiokawa M., Kaname Y., Kambara H., Mori Y., Abe T., Moriishi K., Matsuura Y. (2010). Involvement of Ceramide in the Propagation of Japanese Encephalitis Virus. J. Virol..

[87] Dong E., Du H., Gardner L. (2020). An interactive web-based dashboard to track COVID-19 in real time. Lancet Infect. Dis..

[88] Darquennes G., Le Corre P., Le Moine O., Loas G. (2021). Association between Functional Inhibitors of Acid Sphingomyelinase (FIASMAs) and Reduced Risk of Death in COVID-19 Patients: A Retrospective Cohort Study. Pharmaceuticals.

[89] Le Corre P., Loas G. (2021). Repurposing functional inhibitors of acid sphingomyelinase (fiasmas): An opportunity against SARS-CoV-2 infection?. J. Clin. Pharm. Ther..

[90] Prakash H., Upadhyay D., Bandapalli O.R., Jain A., Kleuser B. (2021). Host sphingolipids: Perspective immune adjuvant for controlling SARS-CoV-2 infection for managing COVID-19 disease. Prostaglandins Other Lipid Mediat..

[91] Becker K.A., Carpinteiro A., Hoffmann M., Pöhlmann S., Kornhuber J., Gulbins E. (2021). Ex vivo assay to evaluate the efficacy of drugs targeting sphingolipids in preventing SARS-CoV-2 infection of nasal epithelial cells. STAR Protoc..

[92] Zhang L.K., Sun Y., Zeng H., Wang Q., Jiang X., Shang W.J., Wu Y., Li S., Zhang Y.L., Hao Z.N. (2020). Calcium channel blocker amlodipine besylate therapy is associated with reduced case fatality rate of COVID-19 patients with hypertension. Cell Discov..

[93] Hoertel N., Sánchez-Rico M., Vernet R., Beeker N., Jannot A.S., Neuraz A., Salamanca E., Paris N., Daniel C., Gramfort A. (2021). Association between antidepressant use and reduced risk of intubation or death in hospitalized patients with COVID-19: Results from an observational study. Mol. Psychiatry.

[94] Caterino M., Gelzo M., Sol S., Fedele R., Annunziata A., Calabrese C., Fiorentino G., D’Abbraccio M., Dell’Isola C., Fusco F.M. (2021). Dysregulation of lipid metabolism and pathological inflammation in patients with COVID-19. Sci. Rep..

[95] Meeusen J.W., Donato L.J., Bryant S.C., Baudhuin L.M., Berger P.B., Jaffe A.S. (2018). Plasma Ceramides. Arterioscler. Thromb. Vasc. Biol..

[96] Edwards M.J., Becker K.A., Gripp B., Hoffmann M., Keitsch S., Wilker B., Soddemann M., Gulbins A., Carpinteiro E., Patel S.H. (2020). Sphingosine prevents binding of SARS-CoV-2 spike to its cellular receptor ACE2. J. Biol. Chem..

[97] Gardner A.I., Haq I.J., Simpson A.J., Becker K.A., Gallagher J., Saint-Criq V., Verdon B., Mavin E., Trigg A., Gray M.A. (2020). Recombinant Acid Ceramidase Reduces Inflammation and Infection in Cystic Fibrosis. Am. J. Respir. Crit. Care Med..

[98] Abu-Farha M., Thanaraj T.A., Qaddoumi M.G., Hashem A., Abubaker J., Al-Mulla F. (2020). The Role of Lipid Metabolism in COVID-19 Virus Infection and as a Drug Target. Int. J. Mol. Sci..

[99] McGowan E.M., Haddadi N., Nassif N.T., Lin Y. (2020). Targeting the SphK-S1P-SIPR Pathway as a Potential Therapeutic Approach for COVID-19. Int. J. Mol. Sci..

[100] Fecchi K., Anticoli S., Peruzzu D., Iessi E., Gagliardi M.C., Matarrese P., Ruggieri A. (2020). Coronavirus Interplay With Lipid Rafts and Autophagy Unveils Promising Therapeutic Targets. Front. Microbiol..

[101] Dadhich R., Kapoor S. (2020). Various Facets of Pathogenic Lipids in Infectious Diseases: Exploring Virulent Lipid-Host Interactome and Their Druggability. J. Membr. Biol..

[102] Sorice M., Misasi R., Riitano G., Manganelli V., Martellucci S., Longo A., Garofalo T., Mattei V. (2020). Targeting Lipid Rafts as a Strategy Against Coronavirus. Front. Cell Dev. Biol..

[103] Steinhart W.L., Busch J.S., Oettgen J.P., Howland J.L. (1984). Sphingolipid Metabolism during Infection of Human Fibroblasts by Herpes Simplex Virus Type 1. Intervirology.

[104] Steinharî W.L., Nicolet C.M., Howland J.L. (1981). Incorporation of ^32^P-Phosphate into Membrane Phospholipids during Infection of Cultured Human Fibroblasts by Herpes Simplex Virus Type 1. Intervirology.

[105] Rivas C.I., Golde D.W., Vera J.C., Kolesnick R.N. (1994). Involvement of the sphingomyelin pathway in autocrine tumor necrosis factor signaling for human immunodeficiency virus production in chronically infected HL-60 cells. Blood.

[106] Papp B., Zhang D., Groopman J.E., Byrn R.A. (1994). Stimulation of human immunodeficiency virus type 1 expression by ceramide. AIDS Res. Hum. Retrovir..

[107] Stenberg R.M. (1996). The Human Cytomegalovirus Major Immediate-Early Gene. Intervirology.

[108] Machesky N.J., Zhang G., Raghavan B., Zimmerman P., Kelly S.L., Merrill A.H., Waldman W.J., Van Brocklyn J.R., Trgovcich J. (2008). Human Cytomegalovirus Regulates Bioactive Sphingolipids. J. Biol. Chem..

[109] Allan-Yorke J., Record M., de Préval C., Davrinche C., Davignon J.-L. (1998). Distinct Pathways for Tumor Necrosis Factor Alpha and Ceramides in Human Cytomegalovirus Infection. J. Virol..

[110] Le Sage V., Cinti A., Amorim R., Mouland A.J. (2016). Adapting the Stress Response: Viral Subversion of the mTOR Signaling Pathway. Viruses.

[111] Grafen A., Schumacher F., Chithelen J., Kleuser B., Beyersdorf N., Schneider-Schaulies J. (2019). Use of Acid Ceramidase and Sphingosine Kinase Inhibitors as Antiviral Compounds Against Measles Virus Infection of Lymphocytes in vitro. Front. Cell Dev. Biol..

[112] Leier H.C., Weinstein J.B., Kyle J.E., Lee J.-Y., Bramer L.M., Stratton K.G., Kempthorne D., Navratil A.R., Tafesse E.G., Hornemann T. (2020). A global lipid map defines a network essential for Zika virus replication. Nat. Commun..

[113] Aktepe T.E., Pham H., Mackenzie J.M. (2015). Differential utilisation of ceramide during replication of the flaviviruses West Nile and dengue virus. Virology.

[114] Gewaid H., Aoyagi H., Arita M., Watashi K., Suzuki R., Sakai S., Kumagai K., Yamaji T., Fukasawa M., Kato F. (2020). Sphingomyelin Is Essential for the Structure and Function of the Double-Membrane Vesicles in Hepatitis C Virus RNA Replication Factories. J. Virol..

[115] Martín-Acebes M.A., Vázquez-Calvo Á., Saiz J.-C. (2016). Lipids and flaviviruses, present and future perspectives for the control of dengue, Zika, and West Nile viruses. Prog. Lipid Res..

[116] Maceyka M., Machamer C.E. (1997). Ceramide Accumulation Uncovers a Cycling Pathway for the cis-Golgi Network Marker, Infectious Bronchitis Virus M Protein. J. Cell Biol..

[117] Tafesse F.G., Sanyal S., Ashour J., Guimaraes C.P., Hermansson M., Somerharju P., Ploegh H.L. (2013). Intact sphingomyelin biosynthetic pathway is essential for intracellular transport of influenza virus glycoproteins. Proc. Natl. Acad. Sci. USA.

[118] Hidari K.I., Suzuki Y., Suzuki T. (2006). Suppression of the biosynthesis of cellular sphingolipids results in the inhibition of the maturation of influenza virus particles in MDCK cells. Biol. Pharm. Bull..

[119] Seo Y.-J., Pritzl C.J., Vijayan M., Bomb K., McClain M.E., Alexander S., Hahm B. (2013). Sphingosine Kinase 1 Serves as a Pro-Viral Factor by Regulating Viral RNA Synthesis and Nuclear Export of Viral Ribonucleoprotein Complex upon Influenza Virus Infection. PLoS ONE.

[120] Xia C., Seo Y.-J., Studstill C.J., Vijayan M., Wolf J.J., Hahm B. (2018). Transient inhibition of sphingosine kinases confers protection to influenza A virus infected mice. Antivir. Res..

[121] Soudani N., Hage-Sleiman R., Karam W., Dbaibo G., Zaraket H. (2019). Ceramide Suppresses Influenza A Virus Replication In Vitro. J. Virol..

[122] Monick M.M., Cameron K., Powers L.S., Butler N.S., McCoy D., Mallampalli R.K., Hunninghake G.W. (2004). Sphingosine Kinase Mediates Activation of Extracellular Signal–Related Kinase and Akt by Respiratory Syncytial Virus. Am. J. Respir. Cell Mol. Biol..

[123] Galvan V., Roizman B. (1998). Herpes simplex virus 1 induces and blocks apoptosis at multiple steps during infection and protects cells from exogenous inducers in a cell-type-dependent manner. Proc. Natl. Acad. Sci. USA.

[124] Sawai H., Okazaki T., Yamamoto H., Okano H., Takeda Y., Tashima M., Sawada H., Okuma M., Ishikura H., Umehara H. (1995). Requirement of AP-1 for ceramide-induced apoptosis in human leukemia HL-60 cells. J. Biol. Chem..

[125] Richard A., Robichaud G., Lapointe R., Bourgoin S., Darveau A., Poulin L. (1997). Interference of HIV-1 Nef in the sphingomyelin transduction pathway activated by tumour necrosis factor-α in human glial cells. AIDS.

[126] Robichaud G.A., Poulin L. (2000). HIV Type 1 nef Gene Inhibits Tumor Necrosis Factor α-Induced Apoptosis and Promotes Cell Proliferation through the Action of MAPK and JNK in Human Glial Cells. AIDS Res. Hum. Retrovir..

[127] Miyake Y., Kozutsumi Y., Nakamura S., Fujita T., Kawasaki T. (1995). Serine palmitoyltransferase is the primary target of a sphingosine-like immunosuppressant, ISP-1/myriocin. Biochem. Biophys. Res. Commun..

[128] Umehara T., Sudoh M., Yasui F., Matsuda C., Hayashi Y., Chayama K., Kohara M. (2006). Serine palmitoyltransferase inhibitor suppresses HCV replication in a mouse model. Biochem. Biophys. Res. Commun..

[129] Amemiya F., Maekawa S., Itakura Y., Kanayama A., Matsui A., Takano S., Yamaguchi T., Itakura J., Kitamura T., Inoue T. (2008). Targeting lipid metabolism in the treatment of hepatitis C virus infection. J. Infect. Dis..

[130] Tatematsu K., Tanaka Y., Sugiyama M., Sudoh M., Mizokami M. (2011). Host sphingolipid biosynthesis is a promising therapeutic target for the inhibition of hepatitis B virus replication. J. Med. Virol..

[131] Ciesek S., Steinmann E., Manns M.P., Wedemeyer H., Pietschmann T. (2008). The suppressive effect that myriocin has on hepatitis C virus RNA replication is independent of inhibition of serine palmitoyl transferase. J. Infect. Dis..

[132] Liu Y., Bochkov Y.A., Eickhoff J.C., Hu T., Zumwalde N.A., Tan J.W., Lopez C., Fichtinger P.S., Reddy T.R., Overmyer K.A. (2020). Orosomucoid-like 3 Supports Rhinovirus Replication in Human Epithelial Cells. Am. J. Respir. Cell Mol. Biol..

[133] Wang J., Guo X., Yang Z., Tan R.X., Chen X., Li E. (2015). Fungal metabolite myriocin promotes human herpes simplex virus-2 infection. Life Sci..

[134] Norred W.P., Wang E., Yoo H., Riley R.T., Merrill A.H. (1992). In vitro toxicology of fumonisins and the mechanistic implications. Mycopathologia.

[135] Izquierdo-Useros N., Naranjo-Gómez M., Archer J., Hatch S.C., Erkizia I., Blanco J., Borràs F.E., Puertas M.C., Connor J.H., Fernández-Figueras M.T. (2009). Capture and transfer of HIV-1 particles by mature dendritic cells converges with the exosome-dissemination pathway. Blood.

[136] Hatch S.C., Archer J., Gummuluru S. (2009). Glycosphingolipid composition of human immunodeficiency virus type 1 (HIV-1) particles is a crucial determinant for dendritic cell-mediated HIV-1 trans-infection. J. Virol..

[137] Amtmann E. (1996). The antiviral, antitumoural xanthate D609 is a competitive inhibitor of phosphatidylcholine-specific phospholipase C. Drugs Exp. Clin. Res..

[138] Luberto C., Hannun Y.A. (1998). Sphingomyelin synthase, a potential regulator of intracellular levels of ceramide and diacylglycerol during SV40 transformation. Does sphingomyelin synthase account for the putative phosphatidylcholine-specific phospholipase C?. J. Biol. Chem..

[139] Meng A., Luberto C., Meier P., Bai A., Yang X., Hannun Y.A., Zhou D. (2004). Sphingomyelin synthase as a potential target for D609-induced apoptosis in U937 human monocytic leukemia cells. Exp. Cell Res..

[140] Sauer G., Amtmann E., Melber K., Knapp A., Müller K., Hummel K., Scherm A. (1984). DNA and RNA virus species are inhibited by xanthates, a class of antiviral compounds with unique properties. Proc. Natl. Acad. Sci. USA.

[141] Müller-Decker K., Amtmann E., Sauer G. (1987). Inhibition of the phosphorylation of the regulatory non-structural protein of vesicular stomatitis virus by an antiviral xanthate compound. J. Gen. Virol..

[142] Mellert W., Amtmann E., Erfle V., Sauer G. (1988). Inhibition of HIV-1 replication by an antiviral xanthate compound in vitro. AIDS Res. Hum. Retroviruses.

[143] Waldeck W. (1990). Antiviral xanthate causes conformational changes in simian virus 40 DNA and chromatin. Oncology.

[144] Villanueva N., Navarro J., Cubero E. (1991). Antiviral effects of xanthate D609 on the human respiratory syncytial virus growth cycle. Virology.

[145] Nguyen A., Guedán A., Mousnier A., Swieboda D., Zhang Q., Horkai D., Le Novere N., Solari R., Wakelam M.J.O. (2018). Host lipidome analysis during rhinovirus replication in HBECs identifies potential therapeutic targets. J. Lipid Res..

[146] Walro D.G., Rosenthal K.S. (1997). The antiviral xanthate compound D609 inhibits herpes simplex virus type 1 replication and protein phosphorylation. Antiviral Res..

[147] Wang H., Maurer B.J. (2006). Fenretinide increased ceramides through progressive de novo synthesis and inhibition of sphingomyelin synthesis in a neuroblastoma cell line. Cancer Res..

[148] Carocci M., Hinshaw S.M., Rodgers M.A., Villareal V.A., Burri D.J., Pilankatta R., Maharaj N.P., Gack M.U., Stavale E.J., Warfield K.L. (2015). The bioactive lipid 4-hydroxyphenyl retinamide inhibits flavivirus replication. Antimicrob. Agents Chemother..

[149] Wang C., Yang S.N.Y., Smith K., Forwood J.K., Jans D.A. (2017). Nuclear import inhibitor N-(4-hydroxyphenyl) retinamide targets Zika virus (ZIKV) nonstructural protein 5 to inhibit ZIKV infection. Biochem. Biophys. Res. Commun..

[150] Pitts J.D., Li P.C., de Wispelaere M., Yang P.L. (2017). Antiviral activity of N-(4-hydroxyphenyl) retinamide (4-HPR) against Zika virus. Antiviral. Res..

[151] Graziano F., Desdouits M., Garzetti L., Podini P., Alfano M., Rubartelli A., Furlan R., Benaroch P., Poli G. (2015). Extracellular ATP induces the rapid release of HIV-1 from virus containing compartments of human macrophages. Proc. Natl. Acad. Sci. USA.

[152] Martín-Acebes M.A., Merino-Ramos T., Blázquez A.-B., Casas J., Escribano-Romero E., Sobrino F., Saiz J.-C. (2014). The Composition of West Nile Virus Lipid Envelope Unveils a Role of Sphingolipid Metabolism in Flavivirus Biogenesis. J. Virol..

[153] Huang Y., Li Y., Zhang H., Zhao R., Jing R., Xu Y., He M., Peer J., Kim Y.C., Luo J. (2018). Zika virus propagation and release in human fetal astrocytes can be suppressed by neutral sphingomyelinase-2 inhibitor GW4869. Cell Discov..

[154] Sakamoto H., Okamoto K., Aoki M., Kato H., Katsume A., Ohta A., Tsukuda T., Shimma N., Aoki Y., Arisawa M. (2005). Host sphingolipid biosynthesis as a target for hepatitis C virus therapy. Nat. Chem. Biol..

[155] Kanj S.S., Dandashi N., El-Hed A., Harik H., Maalouf M., Kozhaya L., Mousallem T., Tollefson A.E., Wold W.S., Chalfant C.E. (2006). Ceramide regulates SR protein phosphorylation during adenoviral infection. Virology.

[156] Laevskaya A., Borovjagin A., Timashev P.S., Lesniak M.S., Ulasov I. (2021). Metabolome-Driven Regulation of Adenovirus-Induced Cell Death. Int. J. Mol. Sci..

[157] Menck K., Sönmezer C., Worst T.S., Schulz M., Dihazi G.H., Streit F., Erdmann G., Kling S., Boutros M., Binder C. (2017). Neutral sphingomyelinases control extracellular vesicles budding from the plasma membrane. J. Extracell. Vesicles.

[158] Chan R., Uchil P.D., Jin J., Shui G., Ott D.E., Mothes W., Wenk M.R. (2008). Retroviruses Human Immunodeficiency Virus and Murine Leukemia Virus Are Enriched in Phosphoinositides. J. Virol..

[159] Lindenbach B.D. (2013). Virion assembly and release. Curr. Top. Microbiol. Immunol..

[160] Amako Y., Syed G.H., Siddiqui A. (2011). Protein Kinase D Negatively Regulates Hepatitis C Virus Secretion through Phosphorylation of Oxysterol-binding Protein and Ceramide Transfer Protein. J. Biol. Chem..

[161] Amako Y., Sarkeshik A., Hotta H., Yates J., Siddiqui A. (2009). Role of Oxysterol Binding Protein in Hepatitis C Virus infection. J. Virol..

[162] Bishé B., Syed G., Siddiqui A. (2012). Phosphoinositides in the Hepatitis C Virus Life Cycle. Viruses.

[163] Roussel É., Lippé R. (2018). Cellular Protein Kinase D Modulators Play a Role during Multiple Steps of Herpes Simplex Virus 1 Egress. J. Virol..

[164] Scheiffele P., Roth M.G., Simons K. (1997). Interaction of influenza virus haemagglutinin with sphingolipid–cholesterol membrane domains via its transmembrane domain. EMBO J..

[165] Pickl W.F., Pimentel-Muiños F.X., Seed B. (2001). Lipid Rafts and Pseudotyping. J. Virol..

[166] Manié S.N., Debreyne S., Vincent S., Gerlier D. (2000). Measles Virus Structural Components Are Enriched into Lipid Raft Microdomains: A Potential Cellular Location for Virus Assembly. J. Virol..

[167] Vincent S., Gerlier D., Manié S.N. (2000). Measles Virus Assembly within Membrane Rafts. J. Virol..

[168] Bavari S., Bosio C.M., Wiegand E., Ruthel G., Will A.B., Geisbert T.W., Hevey M., Schmaljohn C., Schmaljohn A., Aman M.J. (2002). Lipid Raft Microdomains: A Gateway for Compartmentalized Trafficking of Ebola and Marburg Viruses. J. Exp. Med..

[169] Barklis E., Alfadhli A., Kyle J.E., Bramer L.M., Bloodsworth K.J., Barklis R.L., Leier H.C., Petty R.M., Zelnik I.D., Metz T.O. (2021). Ceramide synthase 2 deletion decreases the infectivity of HIV-1. J. Biol. Chem..

[170] Suzuki Y., Sato K., Kiso M., Hasegawa A. (1990). New ganglioside analogs that inhibit influenze virus sialidase. Glycoconj. J..

[171] Imre G. (2020). The involvement of regulated cell death forms in modulating the bacterial and viral pathogenesis. Int. Rev. Cell Mol. Biol..

[172] Henderson G., Peng W., Jin L., Perng G.C., Nesburn A.B., Wechsler S.L., Jones C. (2002). Regulation of caspase 8- and caspase 9-induced apoptosis by the herpes simplex virus type 1 latency-associated transcript. J. Neurovirol..

[173] Danthi P. (2016). Viruses and the Diversity of Cell Death. Annu. Rev. Virol..

[174] Jan J.-T., Chatterjee S., Griffin D.E. (2000). Sindbis Virus Entry into Cells Triggers Apoptosis by Activating Sphingomyelinase, Leading to the Release of Ceramide. J. Virol..

[175] Griffin D.E., Griffin D.E. (2005). Neuronal Cell Death in Alphavirus Encephalomyelitis. Role of Apoptosis in Infection.

[176] Sol N., Le Junter J., Vassias I., Freyssinier J.M., Thomas A., Prigent A.F., Rudkin B.B., Fichelson S., Morinet F. (1999). Possible Interactions between the NS-1 Protein and Tumor Necrosis Factor Alpha Pathways in Erythroid Cell Apoptosis Induced by Human Parvovirus B19. J. Virol..

[177] Perry S.W., Hamilton J.A., Tjoelker L.W., Dbaibo G., Dzenko K.A., Epstein L.G., Hannun Y., Whittaker J.S., Dewhurst S., Gelbard H.A. (1998). Platelet-activating factor receptor activation. An initiator step in HIV-1 neuropathogenesis. J. Biol. Chem..

[178] Haughey N.J., Cutler R.G., Tamara A., McArthur J.C., Vargas D.L., Pardo C.A., Turchan J., Nath A., Mattson M.P. (2004). Perturbation of sphingolipid metabolism and ceramide production in HIV-dementia. Ann. Neurol..

[179] Jana A., Pahan K. (2004). Human Immunodeficiency Virus Type 1 gp120 Induces Apoptosis in Human Primary Neurons through Redox-Regulated Activation of Neutral Sphingomyelinase. J. Neurosci..

[180] Fujinami R.S., Zurbriggen A., Powell H.C. (1988). Monoclonal antibody defines determinant between Theiler’s virus and lipid-like structures. J. Neuroimmunol..

[181] Yamada M., Zurbriggen A., Fujinami R.S. (1990). Monoclonal antibody to Theiler’s murine encephalomyelitis virus defines a determinant on myelin and oligodendrocytes, and augments demyelination in experimental allergic encephalomyelitis. J. Exp. Med..

[182] Pathak S., Illavia S.J., Khalili-Shirazi A., Webb H.E. (1990). Immunoelectron microscopical labelling of a glycolipid in the envelopes of brain cell-derived budding viruses, Semliki Forest, influenza and measles, using a monoclonal antibody directed chiefly against galactocerebroside resulting from Semliki Forest virus infection. J. Neurol. Sci..

[183] Webb H.E., Mehta S., Gregson N.A., Leibowitz S. (1984). Immunological reaction of the demyelinating Semliki Forest virus with immune serum to glycolipids and its possible importance to central nervous system viral auto-immune disease. Neuropathol. Appl. Neurobiol..

[184] Atkins G.J., Mooney D.A., Fahy D.A., Ng S.H., Sheahan B.J. (1991). Multiplication of rubella and measles viruses in primary rat neural cell cultures: Relevance to a postulated triggering mechanism for multiple sclerosis. Neuropathol. Appl. Neurobiol..

[185] Amor S., Webb H.E. (1988). CNS pathogenesis following a dual viral infection with Semliki Forest (alphavirus) and Langat (flavivirus). Br. J. Exp. Pathol.

[186] Gould S.J., Booth A.M., Hildreth J.E. (2003). The Trojan exosome hypothesis. Proc. Natl. Acad. Sci. USA.

[187] Chapuy-Regaud S., Dubois M., Plisson-Chastang C., Bonnefois T., Lhomme S., Bertrand-Michel J., You B., Simoneau S., Gleizes P.-E., Flan B. (2017). Characterization of the lipid envelope of exosome encapsulated HEV particles protected from the immune response. Biochimie.

[188] Sanada T., Hirata Y., Naito Y., Yamamoto N., Kikkawa Y., Ishida Y., Yamasaki C., Tateno C., Ochiya T., Kohara M. (2017). Transmission of HBV DNA Mediated by Ceramide-Triggered Extracellular Vesicles. Cell. Mol. Gastroenterol. Hepatol..

[189] Avota E., Gassert E., Schneider-Schaulies S. (2011). Cytoskeletal Dynamics: Concepts in Measles Virus Replication and Immunomodulation. Viruses.

[190] Gassert E., Avota E., Harms H., Krohne G., Gulbins E., Schneider-Schaulies S. (2009). Induction of Membrane Ceramides: A Novel Strategy to Interfere with T Lymphocyte Cytoskeletal Reorganisation in Viral Immunosuppression. PLoS Pathog..

[191] Mueller N., Avota E., Collenburg L., Grassmé H., Schneider-Schaulies S. (2014). Neutral Sphingomyelinase in Physiological and Measles Virus Induced T Cell Suppression. PLoS Pathog..

[192] Brennan P.J., Brigl M., Brenner M.B. (2013). Invariant natural killer T cells: An innate activation scheme linked to diverse effector functions. Nat. Rev. Immunol..

[193] Juno J.A., Keynan Y., Fowke K.R. (2012). Invariant NKT cells: Regulation and function during viral infection. PLoS Pathog..

[194] Saroha A., Pewzner-Jung Y., Ferreira N.S., Sharma P., Jouan Y., Kelly S.L., Feldmesser E., Merrill A.H., Trottein F., Paget C. (2017). Critical Role for Very-Long Chain Sphingolipids in Invariant Natural Killer T Cell Development and Homeostasis. Front. Immunol..

[195] Fernandez C.S., Kelleher A.D., Finlayson R., Godfrey D.I., Kent S.J. (2014). NKT cell depletion in humans during early HIV infection. Immunol. Cell Biol..

[196] Tessmer M.S., Fatima A., Paget C., Trottein F., Brossay L. (2009). NKT cell immune responses to viral infection. Expert Opin. Ther. Targets.

[197] Raftery M.J., Winau F., Giese T., Kaufmann S.H.E., Schaible U.E., Schönrich G. (2008). Viral danger signals control CD1d de novo synthesis and NKT cell activation. Eur. J. Immunol..

[198] Paquin-Proulx D., Gibbs A., Bächle S.M., Checa A., Introini A., Leeansyah E., Wheelock C.E., Nixon D.F., Broliden K., Tjernlund A. (2016). Innate Invariant NKT Cell Recognition of HIV-1–Infected Dendritic Cells Is an Early Detection Mechanism Targeted by Viral Immune Evasion. J. Immunol..

[199] Opasawatchai A., Matangkasombut P. (2015). iNKT Cells and Their Potential Lipid Ligands during Viral Infection. Front. Immunol..

[200] Brutkiewicz R.R., Yunes-Medina L., Liu J. (2018). Immune evasion of the CD1d/NKT cell axis. Curr. Opin. Immunol..

[201] Bosnjak L., Sahlström P., Paquin-Proulx D., Leeansyah E., Moll M., Sandberg J.K. (2012). Contact-Dependent Interference with Invariant NKT Cell Activation by Herpes Simplex Virus-Infected Cells. J. Immunol..

[202] Reilly E.C., Thompson E.A., Aspeslagh S., Wands J.R., Elewaut D., Brossay L. (2012). Activated iNKT cells promote memory CD8+ T cell differentiation during viral infection. PLoS ONE.

[203] Amador-Molina A., Trejo-Moreno C., Romero-Rodríguez D., Sada-Ovalle I., Pérez-Cárdenas E., Lamoyi E., Moreno J., Lizano M. (2019). Vaccination with human papillomavirus-18 E1 protein plus α-galactosyl-ceramide induces CD8(+) cytotoxic response and impairs the growth of E1-expressing tumors. Vaccine.

[204] Anderson R.J., Li J., Kedzierski L., Compton B.J., Hayman C.M., Osmond T.L., Tang C.-w., Farrand K.J., Koay H.-F., Almeida C.F.D.S.S.E. (2017). Augmenting Influenza-Specific T Cell Memory Generation with a Natural Killer T Cell-Dependent Glycolipid–Peptide Vaccine. ACS Chem. Biol..

[205] Fotouhi F., Shaffifar M., Farahmand B., Shirian S., Saeidi M., Tabarraei A., Gorji A., Ghaemi A. (2017). Adjuvant use of the NKT cell agonist alpha-galactosylceramide leads to enhancement of M2-based DNA vaccine immunogenicity and protective immunity against influenza A virus. Arch. Virol..

[206] Artiaga B.L., Yang G., Hutchinson T.E., Loeb J.C., Richt J.A., Lednicky J.A., Salek-Ardakani S., Driver J.P. (2016). Rapid control of pandemic H1N1 influenza by targeting NKT-cells. Sci. Rep..

[207] Dwivedi V., Manickam C., Dhakal S., Binjawadagi B., Ouyang K., Hiremath J., Khatri M., Hague J.G., Lee C.W., Renukaradhya G.J. (2016). Adjuvant effects of invariant NKT cell ligand potentiates the innate and adaptive immunity to an inactivated H1N1 swine influenza virus vaccine in pigs. Vet. Microbiol..

[208] Artiaga B.L., Yang G., Hackmann T.J., Liu Q., Richt J.A., Salek-Ardakani S., Castleman W.L., Lednicky J.A., Driver J.P. (2016). α-Galactosylceramide protects swine against influenza infection when administered as a vaccine adjuvant. Sci. Rep..

[209] Li K., Luo J., Wang C., He H. (2011). α-Galactosylceramide potently augments M2e-induced protective immunity against highly pathogenic H5N1 avian influenza virus infection in mice. Vaccine.

[210] Kamijuku H., Nagata Y., Jiang X., Ichinohe T., Tashiro T., Mori K., Taniguchi M., Hase K., Ohno H., Shimaoka T. (2008). Mechanism of NKT cell activation by intranasal coadministration of α-galactosylceramide, which can induce cross-protection against influenza viruses. Mucosal Immunol..

[211] Youn H.-J., Ko S.-Y., Lee K.-A., Ko H.-J., Lee Y.-S., Fujihashi K., Boyaka P.N., Kim S.-H., Horimoto T., Kweon M.-N. (2007). A single intranasal immunization with inactivated influenza virus and α-galactosylceramide induces long-term protective immunity without redirecting antigen to the central nervous system. Vaccine.

[212] Kopecky-Bromberg S.A., Fraser K.A., Pica N., Carnero E., Moran T.M., Franck R.W., Tsuji M., Palese P. (2009). Alpha-C-galactosylceramide as an adjuvant for a live attenuated influenza virus vaccine. Vaccine.

[213] Miller D.S., Finnie J., Bowden T.R., Scholz A.C., Oh S., Kok T., Burrell C.J., Trinidad L., Boyle D.B., Li P. (2011). Preclinical efficacy studies of influenza A haemagglutinin precursor cleavage loop peptides as a potential vaccine. J. Gen. Virol..

[214] Lindqvist M., Persson J., Thörn K., Harandi A.M. (2009). The Mucosal Adjuvant Effect of α-Galactosylceramide for Induction of Protective Immunity to Sexually Transmitted Viral Infection. J. Immunol..

[215] Iversen M.B., Jensen S.K., Hansen A.L., Winther H., Issazadeh-Navikas S., Reinert L.S., Holm C.K. (2015). NKT cell activation by local α-galactosylceramide administration decreases susceptibility to HSV-2 infection. Immunobiology.

[216] Huang Y., Chen A., Li X., Chen Z., Zhang W., Song Y., Gurner D., Gardiner D., Basu S., Ho D.D. (2008). Enhancement of HIV DNA vaccine immunogenicity by the NKT cell ligand, α-galactosylceramide. Vaccine.

[217] Singh S., Yang G., Byrareddy S.N., Barry M.A., Sastry K.J. (2014). Natural killer T cell and TLR9 agonists as mucosal adjuvants for sublingual vaccination with clade C HIV-1 envelope protein. Vaccine.

[218] Cox R.G., Erickson J.J., Hastings A.K., Becker J.C., Johnson M., Craven R.E., Tollefson S.J., Boyd K.L., Williams J.V. (2014). Human Metapneumovirus Virus-Like Particles Induce Protective B and T Cell Responses in a Mouse Model. J. Virol..

[219] Kim Y.-J., Han S.-H., Kang H.-W., Lee J.-M., Kim Y.-S., Seo J.-H., Seong Y.-K., Ko H.-J., Choi T.H., Moon C. (2011). NKT ligand-loaded, antigen-expressing B cells function as long-lasting antigen presenting cells in vivo. Cell. Immunol..

[220] Yun S.O., Shin H.Y., Kang C.-Y., Kang H.J. (2017). Generation of antigen-specific cytotoxic T lymphocytes with activated B cells. Cytotherapy.

[221] Kakimi K., Guidotti L.G., Koezuka Y., Chisari F.V. (2000). Natural killer T cell activation inhibits hepatitis B virus replication in vivo. J. Exp. Med..

[222] Ito H., Ando K., Ishikawa T., Nakayama T., Taniguchi M., Saito K., Imawari M., Moriwaki H., Yokochi T., Kakumu S. (2008). Role of Vα14+ NKT cells in the development of Hepatitis B virus-specific CTL: Activation of Vα14+ NKT cells promotes the breakage of CTL tolerance. Int. Immunol..

[223] Ho L.-P., Denney L., Luhn K., Teoh D., Clelland C., McMichael A.J. (2008). Activation of invariant NKT cells enhances the innate immune response and improves the disease course in influenza. A virus infection. Eur. J. Immunol..

[224] Barthelemy A., Ivanov S., Hassane M., Fontaine J., Heurtault B., Frisch B., Faveeuw C., Paget C., Trottein F. (2016). Exogenous Activation of Invariant Natural Killer T Cells by α-Galactosylceramide Reduces Pneumococcal Outgrowth and Dissemination Postinfluenza. mBio.

[225] Wu C.Y., Feng Y., Qian G.C., Wu J.H., Luo J., Wang Y., Chen G.J., Guo X.K., Wang Z.J. (2010). α-Galactosylceramide protects mice from lethal Coxsackievirus B3 infection and subsequent myocarditis. Clin. Exp. Immunol..

[226] Huber S.A., Roberts B., Moussawi M., Boyson J.E. (2013). Slam Haplotype 2 Promotes NKT But Suppresses Vγ4+ T-Cell Activation in Coxsackievirus B3 Infection Leading to Increased Liver Damage But Reduced Myocarditis. Am. J. Pathol..

[227] Exley M.A., Bigley N.J., Cheng O., Tahir S.M., Smiley S.T., Carter Q.L., Stills H.F., Grusby M.J., Koezuka Y., Taniguchi M. (2001). CD1d-reactive T-cell activation leads to amelioration of disease caused by diabetogenic encephalomyocarditis virus. J. Leukoc. Biol..

[228] Mehta A.S., Gu B., Conyers B., Ouzounov S., Wang L., Moriarty R.M., Dwek R.A., Block T.M. (2004). alpha-Galactosylceramide and novel synthetic glycolipids directly induce the innate host defense pathway and have direct activity against hepatitis B and C viruses. Antimicrob. Agents Chemother..

[229] Kornhuber J., Tripal P., Reichel M., Mühle C., Rhein C., Muehlbacher M., Groemer T.W., Gulbins E. (2010). Functional Inhibitors of Acid Sphingomyelinase (FIASMAs): A novel pharmacological group of drugs with broad clinical applications. Cell Physiol. Biochem..

[230] Phillips N. (2021). The coronavirus is here to stay—Here’s what that means. Nature.

[231] Wu B., Huang Y., Braun A.L., Tong Z., Zhao R., Li Y., Liu F., Zheng J.C. (2015). Glutaminase-containing microvesicles from HIV-1-infected macrophages and immune-activated microglia induce neurotoxicity. Mol. Neurodegener..

